# Evaluating CFIR 2.0 in identifying digital twin implementation challenges in healthcare: bridging the dichotomy between engineering and healthcare communities

**DOI:** 10.3389/fdgth.2025.1611225

**Published:** 2025-09-15

**Authors:** Md Doulotuzzaman Xames, Taylan G. Topcu, Sarah H. Parker, Vivian Zagarese, John W. Epling

**Affiliations:** ^1^ Grado Department of Industrial and Systems Engineering, Virginia Tech, Blacksburg, VA, United States; ^2^ Department of Health Systems and Implementation Science, Virginia Tech Carilion School of Medicine, Roanoke, VA, United States; ^3^ Department of Family and Community Medicine, Virginia Tech Carilion School of Medicine, Roanoke, VA, United States

**Keywords:** digital twin, technology implementation, implementation science, CFIR, healthcare systems engineering

## Abstract

**Background:**

Digital twin (DT) technology holds significant promise for healthcare systems (HSs) due to real-time monitoring based on streaming operational data and *a priori* analysis capabilities without interrupting clinical workflows. However, the sociotechnical complexity of HSs presents challenges for effective DT implementation. A dichotomy also exists between the engineering and implementation science (IS) communities regarding DT implementation challenges. This study assesses the efficacy of the updated Consolidated Framework for Implementation Research (CFIR 2.0) in identifying DT implementation challenges, aiming to bridge the knowledge gap between IS and DT communities.

**Methods:**

This study presents findings from a DT implementation case study in a family medicine clinic, an operational healthcare microsystem. It adopts CFIR 2.0 to guide semi-structured interviews with four key stakeholder groups (e.g., family medicine specialists, engineers, organizational psychologists, and implementation scientists). Participants (*N* = 8) were purposively sampled based on their roles in DT implementation. Thematic coding categorized interview data into seven themes: technological, data-related, financial and economic, regulatory and ethical, organizational, operational, and personnel. Thematic data were then cross-analyzed with challenges documented in DT literature to assess how effectively CFIR 2.0 identifies DT implementation challenges.

**Results:**

Challenges were grouped into three categories: (i) shared challenges captured by both IS and DT communities, (ii) CFIR 2.0-identified challenges overlooked in DT literature, and (iii) challenges documented in DT research but not captured through CFIR 2.0-guided interviews. While there was strong overlap between the communities, a formidable gap also remains. CFIR 2.0 effectively identified a diverse set of issues—predominantly in organizational, financial, and operational themes—including many overlooked by the DT community. However, it was less effective in capturing technological and data-related barriers critical to DT performance, such as modeling, real-time synchronization, and sensor reliability.

**Conclusions:**

CFIR 2.0 effectively identifies organizational and operational barriers to DT implementation in healthcare but falls short in addressing technological and data-related complexities. This study highlights the need for interdisciplinary collaboration for the successful transition of emerging DT technologies into practice to maximize their impact on HS efficiency and patient outcomes.

## 1 Introduction

Healthcare systems (HSs) and organizations regularly invest significant financial, technical, and clinical resources into interventions (e.g., new technologies, processes, guidelines) that are aimed at improving care quality and organizational efficiency (1, 2). Nevertheless, a high-quality, evidence-based intervention does not ensure the expected benefits will be realized. Accurate modeling of HSs with explicit consideration of the downstream impact of interventions on daily workflows could facilitate smoother implementation; however, this is challenging due to the inherent complexity of HSs (3–5) and the involvement of human actors at multiple levels (6, 7). Traditional simulations, while useful (8), are time-consuming, resource-intensive, and often fail to capture the dynamic nature of real clinical settings (9, 10). Additionally, policy recommendations generated by traditional simulations do not offer a direct implementation interface into HS operations (11) and their realization is often hindered by sociotechnical barriers (12–14). To that end, emerging digital twin (DT) technology bears great promise (15–17).

DTs offer a compelling alternative to traditional modeling and simulation-based approaches by integrating virtual representations of physical entities with real-time operational data and automated reasoning capabilities (18, 19), enabling continuous and real-time updates (20, 21). Using DTs, HSs could rigorously test new interventions in a low-risk virtual environment without disturbing daily operations and identify associated implementation risks *a priori*. This approach could enhance healthcare leaders' and frontline staff's understanding of the impact of an intervention before implementation. Ultimately, DTs could support the Quintuple Aim (22) as they are documented to enhance predictive accuracy, minimize intervention delays, and inform decisions that could concurrently improve the quality of care (23), provider well-being (24), health equity (25), efficiency of operations (26), and HS sustainability (27).

However, the intricate and dynamic nature of HSs—driven by the complex interplay between biological processes, human decision-making, and technology—presents unique challenges for *implementing DTs into HSs effectively* (16). Given the expected widespread adoption of DTs (16), identifying these challenges within the healthcare setting is crucial. Nevertheless, a dichotomy exists in the literature regarding the documentation of these challenges, specifically between the Implementation Science (IS) community and the engineering community that has been leading the development of DTs.

The IS community recognizes the sociotechnical complexity of HSs and relies on comprehensive frameworks, such as the Consolidated Framework for Implementation Research (CFIR) (28), to identify implementation barriers (29–31). Although CFIR is mainly used for clinical interventions, its application to technological interventions, like DTs, is still nascent (32–34); and it remains to be seen whether it could facilitate the implementation of DTs into practice. Meanwhile, the engineering community that has been spearheading the development of DTs is predominantly concerned with maturing the technology and currently overlooks implementation concerns, particularly those related to the unique contextual setting of HSs. Our objective is to assess CFIR's effectiveness and identify opportunities for knowledge transfer between IS and DT communities. To that end, documenting knowledge gaps between IS and DT research is crucial for expediting DT adoption. This paper addresses this problem at its core by addressing the following research questions (RQs):

 RQ1: How effective is CFIR 2.0 in identifying DT implementation challenges in HSs?
 RQ2: What are the knowledge gaps between the IS and engineering communities on DT implementation, and how can they be bridged?

We explore these questions using a representative DT case study on provider workload in an operational HS microsystem, a family medicine clinic. We used CFIR 2.0 (35) to extract data on DT implementation challenges from key HS stakeholders. We then compare CFIR 2.0 findings with implementation challenges that are currently documented in the engineering literature. We find that CFIR is effective in identifying numerous implementation barriers, including novel ones that are currently overlooked by the engineering community. However, several significant challenges that relate to modeling, connectivity, data fusion, and lifecycle management remained undetected in our case study, despite including key stakeholders from all relevant disciplines. Findings reveal the multifaceted nature of these implementation challenges and the critical role of details in successful DT implementation in HS operations. We discuss how a more integrated approach between these communities could support the implementation process and improve related outcomes.

## 2 Literature review

### 2.1 Digital twins in healthcare systems

Although specific definitions vary based on the application area, a DT could be defined as the combination of a physical system, its virtual representation, and the bilateral data and information flow linking these two (36). Over the past two decades, advances in artificial intelligence (AI), machine learning (ML), and the Internet of Things (IoT) have driven the growth of DT research (19), enabling capabilities like system health monitoring (37), anomaly detection (38), and predictive maintenance (39). While DT applications are used in increasingly more diverse sectors, they are most prevalent in engineering fields such as manufacturing, civil, and aerospace (40).

DT research for HSs has grown rapidly in recent years, focusing primarily on improving patient care (16, 41, 42). A recent systematic review categorizes this body of research into four HS contexts: the patient's body, medical procedures, facilities, and public health (16). DTs within the context of a patient's body are used for monitoring health (43), early diagnosis of diseases (44), aiding rehabilitation (45); and providing personalized treatment by managing biological processes in cells (46) and organs (47), as well as supporting precision medicine through augmented intelligence and patient-specific modeling approaches (48, 49). In terms of medical procedures, DTs are developed to govern medical robots for precision surgery (50), advanced sensors for data collection (51), and wearable exoskeletons for monitoring (52). Similarly, DTs are leveraged to assist robotic surgeries (53, 54), dental procedures (55), and other surgical decision-making (56). In the context of healthcare facilities, DTs enhance operations of hospitals in general (57, 58) or specific HS units such as emergency departments (59, 60) through improved staff scheduling (61) and workflow optimization (60). Other applications include remote patient monitoring (62), mental health management (63), drug development and testing (64), public health management (23), and pandemic monitoring (65).

Nevertheless, the vast majority of this research remains conceptual, and research on successful DT integration into HS practice is nascent (16). This is concerning given the lackluster history of technology implementation in HSs, such as in the case of EHR (66, 67), and the complex socio-technical interactions that constitute HS operations (3, 68). These concerns also motivate this study.

### 2.2 Implementation science frameworks

The Implementation Science (IS) community offers numerous frameworks to guide researchers and practitioners in the adoption, integration, and evaluation of evidence-based interventions in healthcare settings (69). Frameworks like the CFIR (28), the Reach, Effectiveness, Adoption, Implementation, and Maintenance (RE-AIM) (70), the Promoting Action on Research Implementation in Health Services (PARiHS) (71), the Ecological framework, and the Non-Adoption, Abandonment, Scale-up, Spread, and Sustainability (NASSS) framework (72) offer comprehensive lenses to analyze the intertwined factors influencing intervention success (69). These frameworks emphasize the dynamic interplay between the organizational context, external environment, and individual stakeholder attributes, and the interaction of these with intervention characteristics. For example, the CFIR organizes these factors into five domains, offering a structured approach to identifying barriers and facilitators. Similarly, RE-AIM focuses on evaluating the public health impact of interventions by assessing reach, effectiveness, adoption, implementation fidelity, and long-term maintenance, making it particularly useful for balancing internal and external validity.

Here, it is useful to emphasize that these frameworks are not mutually exclusive but rather complementary in terms of addressing different implementation challenges. For instance, the PARiHS highlights the role of evidence, context, and facilitation in driving successful implementation, making it particularly useful in healthcare settings where stakeholder engagement and organizational readiness are critical (73). On the other hand, the NASSS addresses the complexity of scaling health technologies by examining domains such as the condition being treated, the technology itself, and the wider socio-political context, providing insights into why interventions may fail or succeed in real-world scenarios (74). Synergistic use of these frameworks could lead to a more holistic understanding of the factors influencing implementation, enabling to design of implementation strategies that are effective, adaptable, and sustainable in diverse settings (75).

CFIR is a framework designed to identify key factors influencing the implementation of healthcare interventions. It is structured around five domains: intervention characteristics, outer setting, inner setting, characteristics of individuals, and the implementation process. Each of these domains encompasses a wide range of constructs that shape how interventions are adopted, implemented, and sustained within HSs. For instance, in the inner setting domain, constructs such as culture, structural characteristics, and mission alignment can significantly impact the successful adoption of an intervention in a facility. CFIR is often regarded as a suitable framework for assessing complex technology implementation (76, 77).

CFIR 2.0, the framework used in this study, is an updated version of CFIR that refines existing constructs and introduces new ones to better address the complexities of healthcare intervention implementation, particularly in the context of technology adoption. Compared to CFIR, CFIR 2.0 offers a more nuanced understanding of contextual factors, such as the dynamic interplay between organizational culture and external policies, as well as a stronger emphasis on stakeholder engagement and the integration of emerging technologies like digital health tools (35). By doing so, CFIR 2.0 provides a more robust and flexible framework for examining implementation challenges associated with complex interventions such as digital health technologies in healthcare environments (78, 79).

Ideally, an evidence-based intervention in healthcare would be tested in a controlled setting, with variables that are well-defined and vary consistently. While early iterations of DTs may have just these qualities, within healthcare applications, the utility of a DT will be maximized in its ability to mirror the complexity and dynamic nature of HSs. Thus, context is imperative for both accurate DT development and for identifying useful interventions.

With this perspective in mind, we explored appropriate implementation frameworks. There are extensive reviews on the variety and purpose of different implementation frameworks (32, 33, 69). Since the purpose of this study is to describe the process of translating DTs into practice, we are interested in preemptively determining which factors might influence implementation outcomes, such as feasibility, acceptability, adoption, etc. To that end, CFIR 2.0 provides a comprehensive, adaptable structure that articulates relevant factors at different levels in an operational context. CFIR 2.0 examines interactions across five domains, offering a holistic view of implementation dynamics. Further, since it clearly defines and labels the constructs that describe the contextual factors, it could help identify barriers and facilitators for implementation, making it suitable for DT development and implementation (80).

### 2.3 The research gap

Despite DTs' emerging potential, the research on their effective integration into HSs is nascent (16, 81). DTs are inherently complex technologies that encompass interconnected processes, including data collection, real-time processing, predictive analytics, and intelligent decision-making. These multifaceted capabilities make their implementation in healthcare particularly challenging, requiring a structured approach to navigate technical, organizational, and contextual barriers. At the same time, DTs are complex technologies that span data collection, processing, and intelligent decision-making capabilities. However, there is a lack of evidence regarding *how* and *to what extent* existing IS frameworks can help identify DT implementation challenges in healthcare. For instance, so far, there has been only one study that explored this issue (82) that used the NASSS framework (72) in the context of cardiovascular medicine DT. However, while valuable, this study is limited to literature-based evidence and did not incorporate a case study of DT implementation to understand the complexities of daily HS operations. Thus, there is an opportunity to strengthen the connection between the implementation challenges with the broader challenges identified in the engineering community. To address this gap, we explore the applicability of CFIR 2.0 in identifying DT implementation challenges through a real-world case study in a relevant HS context: a family medicine clinic—a critical component of the U.S. healthcare system that serves as the first point of contact for many patients and is often burdened by high workloads (83). We then contrast our findings with the DT literature to highlight knowledge gaps and transfer opportunities.

## 3 Materials and methods

### 3.1 Research methodology

**Figure 1:**
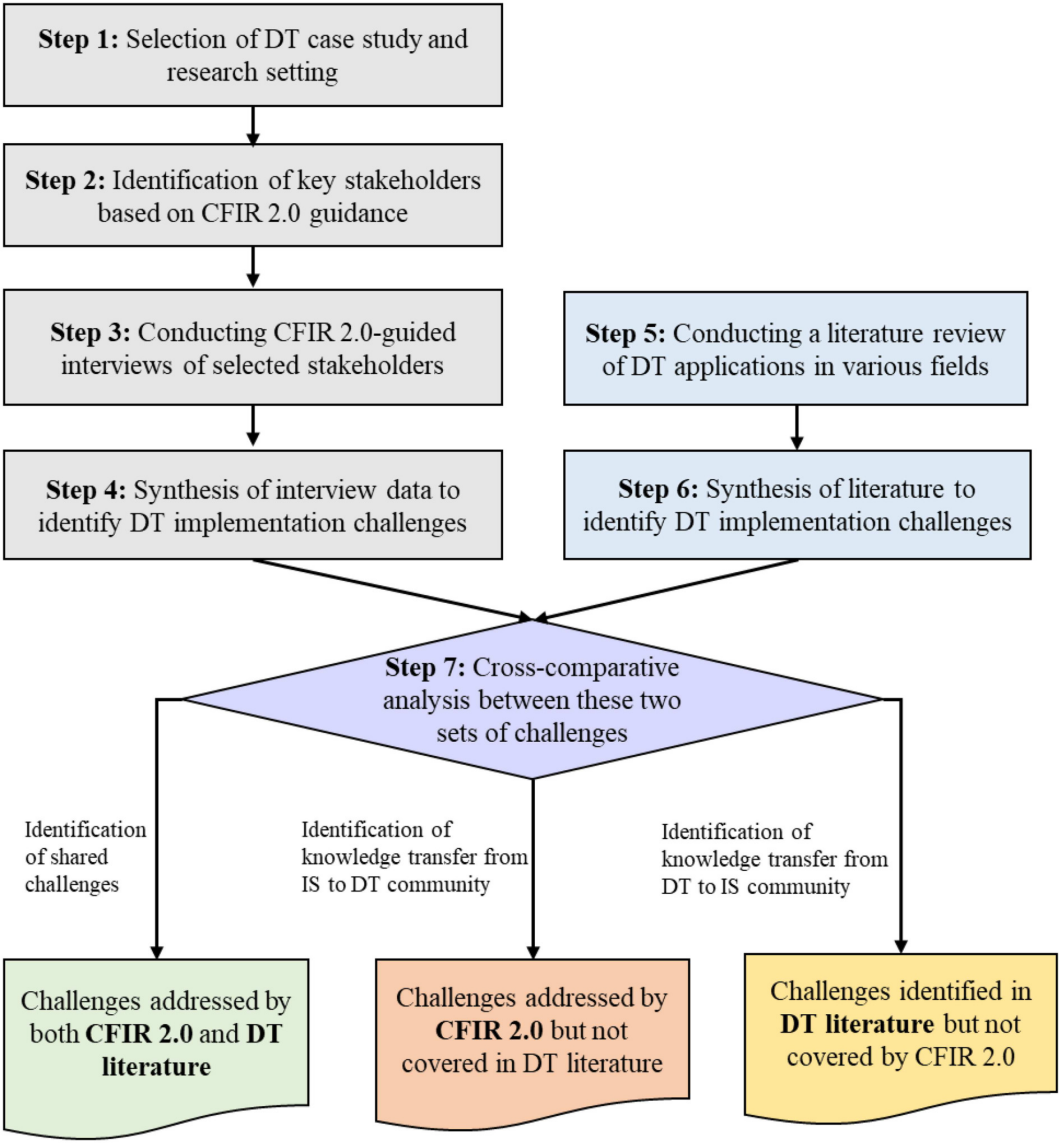
Research framework used in this study.

Figure 1 outlines our approach, which leverages our healthcare DT implementation case study (24), the CFIR 2.0 framework, and a comprehensive evaluation of the DT literature to identify implementation challenges. Our qualitative approach incorporates stakeholder interviews while maintaining reflexivity, and the research paradigm aligns with constructivism/interpretivism. Below, we elaborate on our framework and discuss the case study in Section 3.2.

In Step 1, we present the DT case study and the operational setting in which the technology implementation will be executed. In Step 2, we followed CFIR 2.0 guidance to identify the key stakeholders that have significant expertise and influence over both HS operations and implementation outcomes. In our case, four distinct groups of stakeholders were: organizational psychologists, implementation scientists, engineers, and family medicine specialists.

In Step 3, we conducted interviews with these stakeholders, discussing the specifics of the DT we are aiming to implement. Following a structured protocol guided by the 39 constructs of CFIR 2.0, each of the interviewees was asked to identify the challenges given their specific role in the organization.

In Step 4, we synthesized the interview data and aggregated them through a deduplication process, resulting in a comprehensive set of challenges as identified by the experts.

In Step 5, we complemented these CFIR 2.0-guided findings through a synthesis of the literature on DT research and extracted data on DT implementation challenges. In Step 6, we synthesized the extracted data from the DT literature to identify unique implementation challenges.

Finally, in Step 7, we performed a cross-comparative analysis between the two sets of challenges identified: those obtained from HS stakeholders following CFIR 2.0 guidance and those synthesized from the DT literature. This analysis revealed three distinct groups of challenges: (i) shared challenges that are captured by both CFIR 2.0 and the DT literature, (ii) challenges identified through CFIR 2.0 but not actively considered in the DT literature, and (iii) challenges addressed in the DT literature but were not revealed by our experimental usage of CFIR 2.0. We consider the first group as shared knowledge, while the latter two groups represent knowledge that should be transferred bilaterally between the IS and DT research communities to address the ongoing dichotomy. Note that in Figure 1, the bolded text within each category highlights challenges that correspond to specific sources. We present our findings in Section 4.

### 3.2 Case study and the research setting

#### 3.2.1 The case study: provider workload DT

The case used in this study is a DT to measure and manage provider workload for HSs, with the long-term objective of assisting in burnout mitigation. Burnout in healthcare is a multifaceted issue that undermines providers' mental health, patient care quality, and workforce stability and imposes significant financial costs (81). Thus, effective workload management is key to addressing the root causes of burnout and mitigating its impact (84, 85). While the detailed conceptual model is presented elsewhere (24), a summary is provided here.

**Figure 2:**
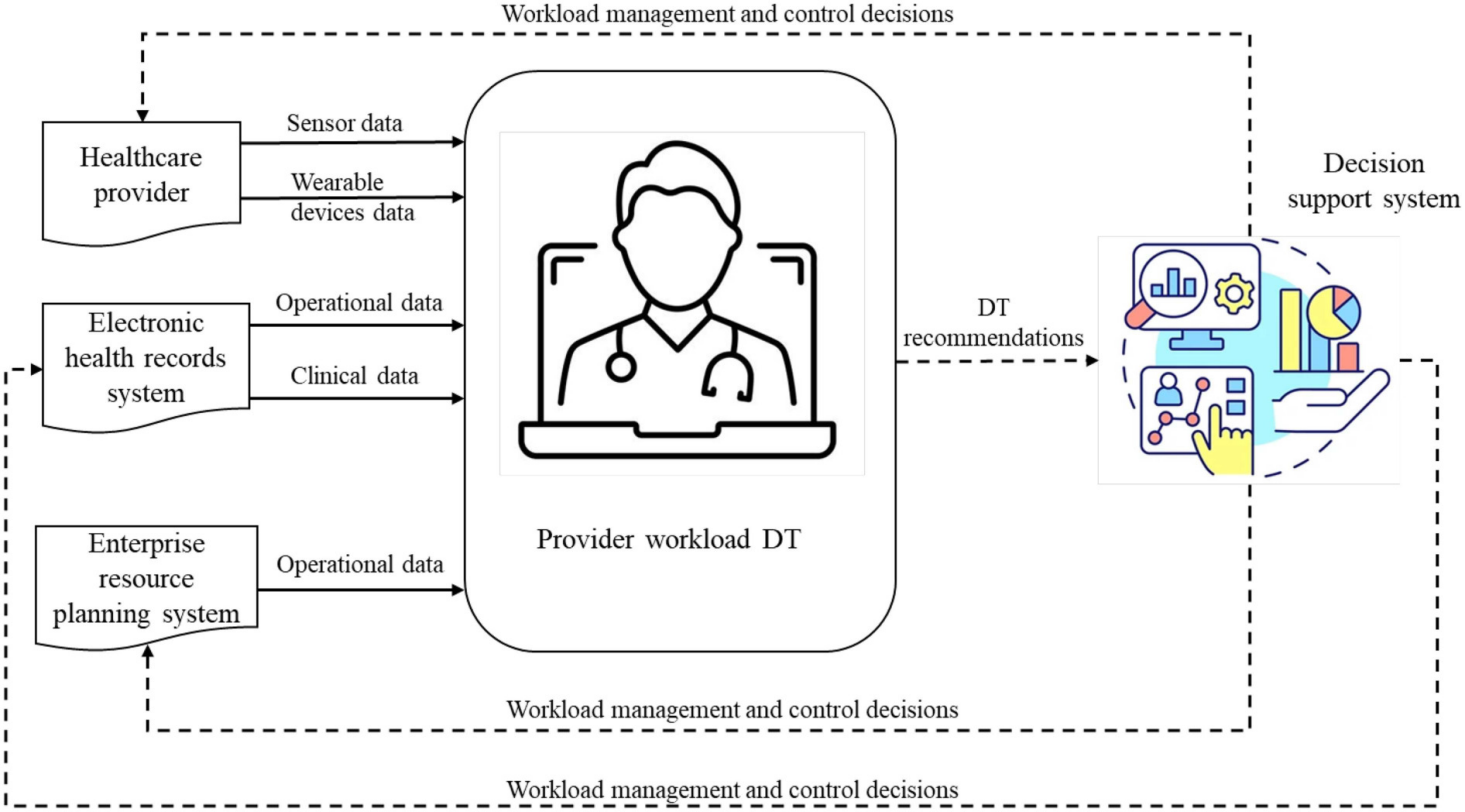
Simplified schematic of the functioning of our representative provider workload DT.

This DT facilitates close to real-time monitoring and management of healthcare provider workload. Figure 2 offers a schematic of its structure, comprising three elements. The *first* element, at the center of the diagram, is a virtual model of physician workload that incorporates an array of data-driven models (e.g., ML, AI). DT generates operational recommendations based on the quantification of physician workload and identifies physicians who are exposed to high risk given their task patterns over time, technological interaction, and human factor concerns. The *second* component is a physical-to-virtual mapping that feeds streaming operational data into the virtual model. This data flow is indicated by solid arrows in Figure 2. This data is collected from various sources, including sensor and wearable devices worn by providers, operational and clinical data from electronic health records (EHR), and other relevant data from enterprise resource planning (ERP) systems. The *third* component is a virtual-to-physical mapping interface, translating DT recommendations into HS operations. This interface is overseen by human decision-makers (e.g., managers) and enables the implementation of recommendations through staffing, process planning, and scheduling decisions. In Figure 2, this information flow is indicated by the dotted arrows. Collectively, this DT provides continuous workload assessment and real-time adjustments, allowing healthcare managers to control and manage workload effectively, ultimately preventing provider burnout.

#### 3.2.2 Research setting: An operational family medicine clinic

Primary care facilities are clinical microsystems (86) that play an integral role in the U.S. healthcare system, serving as the first point of contact for most patients and managing a high volume of patient care situations across a wide spectrum of conditions (83, 87). We chose to conduct our study in a family medicine clinic because primary care physicians represent 29.9% of active physicians in the U.S., with 38.8% specializing in family medicine (88), thus constituting a significant portion of care delivery. Further, an estimated 51% of family physicians in 2022 reported being burned out (89), which aligns the setting well with the proposed case study. Within our chosen observation setting, there are over 50 physicians. This sample is carefully chosen to represent a diverse range of demographics and expertise, ensuring that the DTs reflect the varied experiences and challenges faced by providers across different walks of life.

#### 3.2.3 Stakeholder identification and interviews

To capture diverse perspectives on DT implementation challenges in HSs, four key stakeholder groups were selected following systems engineering best practices (90, 91). These groups included engineers, implementation scientists, organizational psychologists, and family medicine specialists, with two participants in each group for purposive sampling. Engineers were included because they are responsible for designing, implementing, and maintaining the DT system. Implementation scientists were selected for their expertise in overseeing and evaluating implementation efforts, ensuring that best practices are followed. Family medicine specialists, as the primary users of the DT system and the focal point of the study, were crucial in providing insights into its real-world applicability and impact on clinical workflows. Organizational psychologists were included to offer perspectives on human factors, behavioral dynamics, and systemic challenges related to the adoption and integration of DTs in healthcare environments.

While the sample size consisted of two participants per stakeholder group (*N* = 8) and may appear limited in size, this is primarily a concern for studies seeking broad generalizability. In our case, this design was purposefully selected based on the study's focused objective and the contextual constraints of the clinical setting. Empirical work by Hennink & Kaiser (92) suggests that data saturation in qualitative research can often be reached with 9–17 interviews in homogeneous populations. Our participants were deeply embedded in the same organizational context and shared a close understanding of the DT implementation process. Thematic analysis revealed substantial redundancy in responses, indicating that saturation was adequately achieved for our study purpose.

These eight stakeholders were selected not only for their direct involvement in the DT case study but also because they work on-site and have deep firsthand knowledge of the HS's operational intricacies. Their embedded roles within the clinic provided them with a comprehensive understanding of the challenges and facilitators affecting DT implementation. To maintain a focused scope, certain groups were not included in the study. For instance, while nurses play a critical role in patient care, we opted to exclude them because family medicine specialists, who work closely with them, were well-positioned to capture their perspectives as part of the broader clinical workflow. The characteristics of the selected participants are summarized in Table 1 below.

**Table 1:** Characteristics of the interview participants in our study.

Stakeholder group	Number of participants	Average years of experience	Highest level of education
Organizational psychologist	2	10 years	PhD
Implementation scientist	2	20 years	PhD
Engineer	2	10 years	PhD; MS
Family medicine specialist	2	25 years	MD

To collect the data, we conducted semi-structured face-to-face interviews guided by CFIR 2.0 constructs, enabling the exploration of specific challenges relevant to each stakeholder group while maintaining consistency across interviews. This approach facilitated the collection of rich, context-specific data from individuals actively involved in HS operations and integral to the DT implementation process.

Data analysis followed a thematic coding approach. Initially, interview transcripts were reviewed independently by two researchers to ensure comprehensive familiarity with the content. Open coding was employed to identify preliminary codes, which were subsequently refined through axial coding to establish overarching themes and sub-themes. To enhance rigor, the researchers engaged in regular debriefing sessions to discuss coding discrepancies and consensus-building discussions to resolve disagreements. To ensure the credibility and trustworthiness of the data analysis, “member checking” was conducted, wherein preliminary findings were shared with participants to verify the accuracy and relevance of the interpretations. This process helped confirm that the identified themes accurately reflected participants’ perspectives. While the data were not blinded during analysis, coders maintained an awareness of potential biases and engaged in reflexive discussions to mitigate their influence.

To enhance transparency and ground the thematic findings, we incorporated illustrative quotes from participants throughout the results section. These quotes were selected based on their clarity, thematic alignment, and stakeholder relevance. We prioritized excerpts that exemplified frequently occurring patterns or provided particularly vivid articulation of a sub-theme. Divergent or conflicting perspectives, such as when stakeholders offered contrasting views on a particular challenge, were coded alongside convergent views and included in the thematic structure without exclusion.

## 4 Results

**Figure 3:**
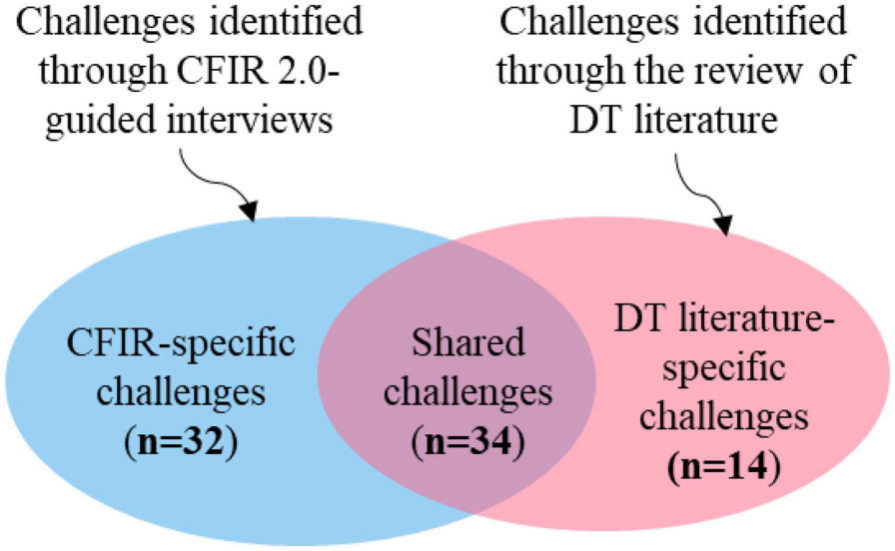
A Venn diagram showing the overlap among the challenges.

In our case study of provider workload DT, we identified a total of 80 implementation challenges through a comprehensive review of DT literature and the CFIR 2.0 framework-guided interviews. We performed a cross-comparative analysis of these datasets, grouping the challenges into three categories, as shown in Figure 3. Of the 80 challenges, 66 were identified through CFIR 2.0-guided interviews (see Supplementary Table 1). Of these, 34 are also recognized in the DT literature. Below is an overview of the findings:

*Shared challenges (n* *=* *34):* Recognized in both CFIR 2.0-guided interviews and DT literature.

*CFIR-specific challenges (n* *=* *32):* Identified through CFIR 2.0 interviews but overlooked in DT literature, highlighting the need for knowledge transfer from IS to DT research community.

*DT literature-specific challenges (n* *=* *14):* Identified only in DT literature, raising concerns about CFIR 2.0's ability to fully capture DT implementation complexities. These represent knowledge transfer opportunities from DT to IS community.

To render it easier to digest for the broader audience, we organized the 80 challenges into seven overarching themes: technological, data-related, financial and economic, regulatory and ethical, organizational, operational, and personnel. These apply to all three challenge groups thus we provide their definitions below:

i. *Technological challenges:* Issues with DT development, integration, and scalability.
ii. *Data-related challenges:* Difficulties in data acquisition, management, quality, and secure exchange across platforms.
iii. *Financial and economic challenges:* Cost-related barriers, including investment, maintenance, return on investment (ROI) uncertainties, and budget constraints.
iv. *Regulatory and ethical challenges:* Legal, regulatory, and ethical concerns, such as patient privacy and healthcare compliance.
v. *Organizational challenges:* Structural and cultural barriers within healthcare institutions.
vi. *Operational challenges:* Practical challenges in testing, training, and integrating DTs into existing workflows.
vii. *Personnel challenges:* Human resource-related barriers affecting DT adoption and integration.

Next, we discuss our findings in detail.

### 4.1 Shared implementation challenges

This section covers overlapping challenges with the DT literature. These challenges, summarized in Table 2, span all seven themes introduced earlier. Here, we note that while some of these challenges appear frequently in DT literature, others—like usability issues, data fragmentation, data governance, and collaboration barriers—are rarely discussed, each appearing in only one reviewed article.

**Table 2:** DT implementation challenges addressed in DT literature and also identified through CFIR 2.0-guided interviews.

Theme	Shared challenges
Technological	• Validation and verification (41, 93, 94, 103) • Standardization (93, 95, 96) • Usability issues (97) • Interoperability (20, 93, 98, 103–105) • Testing and evaluation (100) • Human-work interaction design (106) • Infrastructure and workflow integration (99, 107) • Scalability (23, 41, 103) • Data fragmentation/Siloed data sources (108) • Performance concerns (101, 102)
Data-related	• High real-time data needs (17, 82, 109) • Data integration/fusion (82, 103, 105, 107, 108, 110–112) • Data accessibility (108) • Data privacy (16, 17, 20, 23, 82, 96–99, 103, 105, 107, 108, 111, 113, 114) • Data security (16, 17, 20, 23, 82, 96–99, 103, 107–109, 113, 114) • Data governance (105) • Data ownership (16, 17, 20, 109)
Financial and economic	• High initial cost (82, 115) • Willingness for upfront investment (82, 107)
Regulatory and ethical	• Legal issues (41, 93, 103, 116) • Regulatory compliance (23, 41, 82, 103, 105, 107, 109) • Ethical issues (16, 20, 23, 25, 82, 93, 97, 98, 103, 107, 109, 112, 116–121)
Organizational	• Organizational inertia/resistance to change (100,122,123) • Organization's technology readiness (17, 122, 124) • Collaboration and communication barriers (125)
Operational	• Lack of training and support infrastructure (41, 95) • Trust and transparency in implementation (96, 97, 113) • Quantification of benefits and outcomes (123)
Personnel	• Lack of expertise (82, 101, 122, 126) • Individual's commitment to implementation (126) • Providers’ perception of the technology/Technophobia among the older generation (103) • Fear of added workload (82, 100) • Individual's inertia/resistance to change (100, 122, 123) • Lack of understanding of the technology (41, 95)

Below, we discuss these shared challenges and their implications for DT implementation in healthcare.

*Technological challenges* are crucial to DT implementation because they directly impact the functionality, reliability, and integration of the technology within existing systems. Among these, verification and validation issues are critical, as inaccurate DTs lead to poor decision-making (93, 94). Additionally, the lack of standardization across DT frameworks hampers interoperability, creating silos that hinder integration with existing systems (95, 96). For instance, this was echoed in the following stakeholder quotes:

“… Data remains siloed. Scheduling data, provider demographic and wellbeing data, patient outcome data, and EPIC operations data are all separate.”—Family medicine specialist #2.

“… There are several different data platforms that could be needed as inputs and outputs. Aligning these inputs for time and subject consistency is challenging. Likewise, translating the DT outputs back into the non-unified software is challenging.”—Implementation scientist #1.

Usability concerns further exacerbate this fragmentation, particularly for non-technical users (97). Interoperability and infrastructure integration issues may require substantial organizational overhauls (98, 99). Furthermore, testing and evaluation concerns are particularly challenging, making it difficult to assess the accuracy and reliability of DTs in real-world conditions (100). Performance issues, particularly in scalability and real-time processing, highlight the gap between the theoretical potential of DTs and their practical implementation (101, 102). Addressing these diverse technological challenges is critical for ensuring DTs can reliably be integrated into HS and enhance healthcare outcomes.

*Data-related challenges* are intrinsically linked to the successful implementation of DTs, as their efficacy is contingent upon the availability and quality of data. The demand for massive real-time data inputs applies significant pressure on HSs, which may not be readily equipped with a suitable data infrastructure to supply the necessary demand (82, 109).

“… To accurately represent the complex human-system interactions, a very large amount of data over a long period of time must be included in the initial model build.”—Engineer #1.

Moreover, the fusion of disparate data sources presents additional challenges that can undermine the coherence of DT outputs (105, 110). Data accessibility is another issue that is often exacerbated by proprietary software, security controls, and organizational silos (108).

“… Accessible data limits the ability of the digital twin to consider the relationship between insurance benefits and regulatory considerations (including publicly reported measures such as LeapFrog, U.S. News, and CMS Five-Star program) with the healthcare delivery entity.”—Family medicine specialist #1.

Perhaps the most concerning are the challenges related to data privacy and security. HSs inherently deal with sensitive information and are particularly vulnerable to data breaches, making robust security measures non-negotiable (113, 114). Effective data governance and clear ownership protocols are essential to mitigate associated risks and ensure accountability (20, 105).

*Financial and economic challenges* present another barrier, particularly the high initial costs of DT deployment, which include software, hardware, and skilled personnel. This burden could be particularly daunting for small and medium-sized healthcare organizations (82, 115). This financial burden is compounded by the uncertainty surrounding the ROI, which can deter organizations from committing to DT investments.

“… The technology should improve operations and thus pay for itself. Without clear financial returns, the willingness of healthcare organizations to invest remains uncertain.”—Organizational scientist #2.

*Regulatory and ethical challenges* are critical to DT implementation and need to be considered proactively as they can lead to legal repercussions, compromised patient safety, and loss of public trust. Legal issues such as liability and intellectual property rights raise significant concerns, particularly when decisions will be directly based on DT outputs without human intervention (41, 116). Ethical concerns demand careful consideration, including potential biases in algorithmic decision-making and the broader societal implications of DTs such as equitable access to technology. These issues can lead to serious consequences, such as misdiagnoses or inappropriate treatment plans if not rigorously addressed (25, 118). For example, biases in algorithmic decision-making could result in unequal treatment recommendations across different patient demographics, undermining trust in the technology and the broader HS. Additionally, failure to comply with regulatory standards can result in legal repercussions and damage public trust, rendering healthcare providers and patients reluctant to adopt these technologies (23, 109).

*Organizational challenges* are frequently overlooked but influence the success of implementation efforts. Organizational inertia, or resistance to change, is a formidable barrier to the adoption of DTs (100).

“… The current state is well-established and unlikely to change without significant external pressure.”—Organizational scientist #1.

This resistance is often rooted in the “fear of the unknown” and concerns about the disruption of established workflows. Additionally, the organizational readiness to adopt new technologies, including the necessary IT infrastructure and culture, is crucial for successful DT implementation (124). Collaboration and communication barriers further complicate this process, as the successful deployment of DTs often requires cross-disciplinary cooperation, which is difficult to achieve in siloed environments (125).

*Operational challenges* reflect the day-to-day realities of implementing DTs in healthcare. Lack of training and support infrastructure hinders effective use, especially in organizations new to DT technologies (41).

Building trust in DTs is another operational challenge, particularly given the high stakes associated with clinical decision-making (113). In healthcare settings, errors stemming from model misjudgments can have serious consequences for patient safety and outcomes. Therefore, transparency into how DTs generate outputs is not just a technical concern but a critical trust-building mechanism for clinicians and administrators. However, this transparency is notoriously difficult to establish for most ML and AI-based models, which often operate as “black boxes” (127). Importantly, ensuring that DTs are positioned as tools that augment rather than replace clinical judgment is essential for promoting trust and acceptance among providers (48). Moreover, quantifying the benefits and outcomes of DT deployment is also crucial for ongoing support, yet difficult to measure reliably (123).

Finally, *personnel challenges* are human resources-related barriers that primarily revolve around the availability, expertise, and attitudes of personnel responsible for deploying and maintaining these systems. The specialized skills required for successful DT implementation are scarce, making it difficult to find and retain talent (101). Moreover, similar to most healthcare interventions, the success of DT projects is closely tied to the commitment of individuals within the organization, particularly those in leadership or key technical roles (126).

“… Key stakeholders essential for validation and implementation may be reluctant to dedicate their own time or their team's time to testing, troubleshooting, and deployment, hindering the implementation process.”—Implementation scientist #2.

Perceptions of DT technology among healthcare providers further compound these challenges. Technophobia, especially among older staff, can significantly hinder DT adoption (103). This may be influenced by previous personal experiences with new technology implementation in healthcare, such as EHR, which is often perceived as challenging and negative (128–130). Additionally, fears of added workload and resistance to change are common in environments where established operational practices are deeply ingrained (82, 100). A lack of understanding of DTs could exacerbate these issues, leading to mistrust and reluctance to engage (41). Addressing these personnel challenges is crucial for ensuring that DTs are not only technically successful but also fully embraced and sustained by the people who will use them.

### 4.2 Knowledge transfer from IS community to DT research community

Our CFIR 2.0-guided interviews with key stakeholders revealed 32 novel challenges that are not currently addressed in the DT literature. These challenges encompass all categories except data-related issues, with organizational challenges being more common, accounting for nearly half (*n* = 15) of the identified problems. This likely reflects the insights of stakeholders directly involved in the implementation process, including organizational psychologists, implementation scientists, and family medicine specialists; that are often overlooked within the engineering community during early-stage technology maturation and treated as a later-stage consideration. Detailed in Table 3, these challenges highlight factors that could affect the successful DT implementation, emphasizing the need for knowledge transfer from the IS community to the DT community. Below, we elaborate on these challenges. Notably, no new data-related or regulatory and ethical challenges emerged from the CFIR 2.0-guided interviews.

**Table 3:** DT implementation challenges identified through CFIR 2.0-guided interviews, however, not addressed in DT literature.

Theme	CFIR-specific challenges
Technological	• Time-scale dependency of DT performance • Model latency and timeliness • User-specific personalization
Data-related	N/A
Financial and economic	• Unclear/intangible benefits • Lack of immediate benefits • ROI uncertainty
Regulatory and ethical	N/A
Organizational	• Free trialability • Lack of established best practice guidelines • Pressure for immediate operational performance • Key performance indicators (KPIs) tracking and management • Resilience to low-frequency, high-impact disruptions • Alignment with organizational goals and stakeholder perceptions/Organization prioritizing other outcomes • Misalignment of outcomes and incentives • Stakeholder engagement • Identification of the need/problem • Cultural emphasis on reactive work and reporting over foundational system issues • Differences in leadership styles • Presence of shadow influencers • Value communication • Change fatigue • Effective reflection, evaluation, and feedback mechanisms
Operational	• Lack of generalizability • Need for integrated decision support systems • Reversibility of operational decisions • Availability of resources to implement operational changes • Scope/adaptability management in a dynamic landscape
Personnel	• Staffing shortage • Lack of understanding of perceived benefits • Provider schedule constraints for technology learning • Differences in motivation levels among individuals/groups • Trust in DT developers/vendors • Lack of prior research/Lack of evidence in human-in-the-loop systems

*Technological challenges* such as time-scale dependencies, model latency, and personalization illustrate the nuanced complexities of DT performance that remain underexplored in the engineering community. Time-scale dependency refers to ensuring DTs function accurately across different time frames—ranging from rapid, short-term changes (e.g., vital sign fluctuations) to longer-term shifts (e.g., chronic disease progression)—an inherent characteristic of healthcare data. Model latency, is another key concern, as it may lead to delays in DT responses and compromise the relevance of DT-generated recommendations.

“… Substantial delays exist between data relationships, such as the time required to finalize revenue, billing, and coding charges, followed by additional lags before their integration into data warehouses like Vizient.”—Family medicine specialist #1.

Furthermore, the need for DTs to be personalized to individual provider or patient needs is often overlooked. Personalization is crucial because healthcare environments are highly variable, and “one-size-fits-all” solutions may fail to account for unique physiological, behavioral, or workload differences, thereby limiting the effectiveness and precision of DT interventions. A relevant participant quote regarding the DT personalization challenge is as follows:

“… The challenge with digital twins in healthcare is making them personalized enough to be useful without being too complex to implement. A one-size-fits-all approach won't work, but too much customization can slow things down and create integration issues.”—Engineer #2.

*Financial and economic challenges*, like unclear or intangible benefits and the lack of immediate returns, pose additional barriers to DT implementation. Our interviews repeatedly reflected concerns about justifying the investment in DT technology in the notoriously resource-constrained healthcare sector, which may require demonstrating clear, tangible outcomes. The difficulty in showcasing immediate benefits, coupled with uncertainty around ROI, often deters organizations from committing to DT projects. These issues reinforce the need for detailed, upfront cost-benefit analyses, especially when the demand for short-term results overshadows the importance of long-term goals.

*Organizational challenges* identified through CFIR 2.0 highlight the intricate nature of healthcare environments and the importance of insider perspectives. The pressure from top management for quick results can create obstacles to DT implementation, as the urgency for “quick wins” often leads to hasty adoption efforts that neglect long-term considerations essential for sustainable operations.

“… There's always pressure from leadership to show quick wins. They want results yesterday, but when you rush the process, you miss the foundation that makes the whole system sustainable in the long run.”—Organizational psychologist #1.

Additionally, the absence of established best practice guidelines complicates the navigation of DT complexities, further hindering effective and lasting implementation. Effective monitoring of KPIs, aligning DT projects with organizational goals, and fostering stakeholder engagement are all vital to success. One direct quote from an implementation scientist that captures the challenge of stakeholder engagement is as follows:

“… If you don't engage stakeholders early and often, you end up building a solution no one really wants or needs. The priorities keep shifting, and without that input, you're always playing catch-up.”—Implementation scientist #2.

Additionally, organizational culture plays a vital role, with factors like change fatigue, varying leadership styles, and the influence of “shadow stakeholders” complicating the adoption process. Moreover, healthcare organizations can request free trials from vendors before committing to DT technology, which adds complexity to implementation by potentially causing disruptions during the transition from trial systems to fully integrated solutions. These challenges highlight the importance of organizational readiness and leadership support necessary to sustain organizational change.

*Operational challenges* present substantial barriers, particularly regarding generalizability and resource availability. The adaptability of DTs to various healthcare contexts is a great technical perk but a major implementation concern as it raises questions about appropriate verification and validation strategies (131). Striking the balance between generalizability and over-fitting a specific operational context remains a key research challenge. Without this calibration flexibility, DTs risk delivering inconsistent results across different scenarios. Additionally, human decision-makers in the loop i.e., the healthcare managers and physicians, require transparent decision support systems that allow them to retain oversight and, if necessary, reverse DT-generated decisions. The scarcity of resources, both time and funding, further complicates the operationalization of DTs, making it difficult for organizations to fully capitalize on their potential. Moreover, scope management is another crucial operational challenge as identified from the participant's quote below:

“… Managing the scope of the digital twin is going to be really challenging. There's always this temptation to include everything about the healthcare system, but that's just not practical. Even if you start with a clear focus, it might not match what stakeholders actually need—or their priorities could shift over time.”—Family medicine specialist #2.

*Personnel challenges* identified through CFIR 2.0 range from staffing shortages to a lack of understanding of DT benefits. Due to the complexity of DT technology, adopters could struggle to see its direct impact on workflows or patient care. Additionally, already overburdened healthcare providers may view this new technology as an additional workload rather than a solution. The lack of time and motivation to engage with DTs can further impede adoption, especially when varying motivation levels among staff lead to uneven integration into daily practice. These issues underscore the need for comprehensive training and support systems to bridge the gap between technology and users. Moreover, the absence of prior research and evidence in real-world contexts could lead to skepticism, making it more difficult for stakeholders to trust DTs. Building this trust requires not only demonstrating DT efficacy but also ensuring transparency in their development and implementation—a fruitful area for further exploration, given its role in healthcare.

### 4.3 Knowledge transfer from DT research community to IS community

Our cross-comparative analysis shows that while CFIR 2.0 is effective in identifying many novel challenges in DT implementation within healthcare, it is not an exhaustive mechanism, particularly missing some well-identified issues recognized by the DT community. As outlined in Table 4, these primarily relate to technological or data-related challenges. It's important to note here that in several categories of challenges—financial, regulatory, operational, and personnel—CFIR 2.0 effectively captured the issues already well-documented by engineers.

**Table 4:** DT implementation challenges addressed in DT literature but not identified through CFIR 2.0-guided interviews.

Theme	DT literature-specific challenges
Technological	• DT modeling (16, 23, 93, 95, 113, 121) • DT twinning/Connectivity (16, 23, 82) • High computational and data storage demands (41, 107, 109, 111) • Model accuracy/fidelity (107, 112) • Sensor damage/failure (82)
Data-related	• Data quality (23, 41, 93, 94, 111) • Data integrity (93, 107) • Data availability (121) • Data collection (16, 94, 112) • Data synchronization (16, 23)
Financial and economic	N/A
Regulatory and ethical	N/A
Organizational	• New business models (95) • Difference between industry and academia DT implementation (132)
Operational	N/A
Personnel	N/A

This highlights an opportunity for knowledge transfer from the DT research community to the IS community and emphasizes the limitations of CFIR 2.0 in addressing challenges specific to emerging technologies like DTs. Below, we elaborate on these challenges and discuss why they are crucial for successful DT implementation.

*Technological challenges* are extensively discussed in the DT literature but were not revealed in our CFIR 2.0-guided interviews, even though specialist engineers were included as key stakeholders. This finding is noteworthy as healthcare organizations increasingly adopt technology within an already intricate and often inefficient socio-technical system. One of these challenges is effective DT modeling, which pertains to creating accurate digital representations of physical systems. Here, the complexity of modeling varies based on the system's nature and the desired fidelity, which in turn impacts DTs' efficacy (16). Sophisticated analytical approaches are needed to ensure sufficiently representative models of real-world processes (133); however, this was not revealed in our CFIR 2.0-guided interviews (93, 95, 113, 121). This finding highlights a limitation in CFIR 2.0, which lacks specific constructs to capture the nuances of DT modeling and its influence on desired implementation outcomes. As a result, healthcare stakeholders could often assume that modeling is well-executed, prioritizing generalizability and practical application while overlooking the complexities of the modeling process that are critical to achieving successful implementation. Another overlooked issue is DT twinning/connectivity, perhaps one of the most pressing challenges studied in the DT literature but not identified through CFIR 2.0. Connectivity between physical and virtual twins (P2V and V2P) is essential for compatibility and real-time data transfer. Any lag or disconnection can lead to misinformed decisions, hindering DT effectiveness (16, 23, 82).

Computational and data storage demands are challenges that are studied extensively in DT literature however were not explicitly recognized in our CFIR 2.0-guided interviews. DTs require substantial computational power and dynamic storage to process large datasets and run simulations (41, 107, 109, 111). This affects the feasibility and scalability of DTs, especially in resource-limited settings. Model accuracy and fidelity are also crucial, as they ensure reliability in predicting real-world scenarios (107, 112). Neglecting these could undermine trust and the eventual practical adoption of DTs.

In addition, sensor damage and failure represent significant hurdles, particularly since CFIR 2.0 does not address failure modes or the long-term sustainability of technological interventions. Sensors, key to DT data collection, are external interfaces that are vulnerable to environmental damage and degradation (134, 135). Their failure disrupts data collection, leading to erroneous outputs. For instance, sensor malfunction, caused by degradation, calibration issues, or environmental factors, can lead to missing or erroneous values. Separately, changes to EHR data formats can disrupt data extraction pipelines, creating incompatibilities that affect the continuity of the DT data flow (82). This underscores the need for robust sensor management and maintenance strategies, an area CFIR 2.0 does not capture.

Additionally, *data-related challenges* such as data collection and synchronization were not captured by CFIR 2.0. These involve sourcing data from various distributed, heterogeneous sources such as medical devices, EHR, and wearables (16, 23, 94, 112). Integrating these diverse data sources requires careful coordination of timing, frequency, and standardization. Accurate timestamps are crucial to establish clear temporal relationships between variables, while managing collection intervals and frequencies ensures smooth data synchronization.

Finally, some *organizational challenges* were also not identified by CFIR 2.0. For instance, developing appropriate business models is documented to be a vital component for assessing the economic and operational viability of DTs (95). Moreover, in industry, DT implementations typically use proprietary software tailored for DT development, whereas academic research relies on general-purpose simulation tools. This difference may contribute to a growing divide between industrial and academic DT implementations (132).

## 5 Discussion

### 5.1 Principal findings

DTs are advancing and transitioning into practice at an astonishing pace. For reference, the global DT market is projected to grow by 50% annually and reach ∼$195B by 2030 (136). Healthcare is expected to be a leading driver of this growth, rendering documentation of DT implementation challenges in HSs critical, particularly given the mixed success of past technology implementations in this sector (81, 128–130). Nevertheless, there is a dichotomy between the DT community that is leading the maturation of this exciting technology and the IS community that is concerned with the transition of innovative approaches into practice. To that end, this study presented results from a case study on a conceptual provider workload DT to be implemented within a real-world clinical microsystem. Leveraging the CFIR 2.0 framework, we conducted interviews with key stakeholders to identify implementation challenges and compared them against those documented in the DT literature. This study reports two main findings: (i) CFIR 2.0's ability to identify DT implementation challenges and (ii) the opportunities for knowledge transfer between IS and DT research communities. We summarize these findings in five key points.

First, there was a notable overlap between issues documented in the DT communities and those we were able to identify through CFIR 2.0-guided interviews, albeit with differences in nuance and priority. This suggests that while CFIR 2.0 is not specifically designed for technology implementation, it is effective in identifying DT implementation challenges, especially when a comprehensive group of stakeholders is engaged methodically. This strength likely stems from the consolidated and generalized nature of the framework's constructs, building confidence in the broader applicability of the framework to various DT applications, and quite possibly similar emerging technologies.

Second, CFIR 2.0 sheds light on organizational and financial issues that are consistently overlooked by the DT community, and are essential for a successful implementation. These challenges were both numerous and diverse, suggesting that CFIR 2.0 could be useful given the versatility of DTs, while concurrently calling for a more explicit consideration of these issues within the DT community. However, it is important to note that while CFIR 2.0 helps identify these challenges, it does not offer solutions, hinting at an opportunity for collaboration between the DT and IS communities to address these obstacles.

Third, and perhaps more relevant for the IS community, we found that CFIR 2.0 does not adequately address several critical technological and data-related challenges that are well-documented in the DT community. This limitation posits that CFIR 2.0 could benefit from certain modifications to capture these issues, particularly in the *Innovation* and *Implementation Process* domains. While this paper does not aim to propose a CFIR 3.0, our findings indicate that CFIR 2.0 should be used with caution, recognizing its limitations—particularly when applied to guide the implementation of advanced technologies.

Fourth, our findings highlight the value of using structured yet adaptable frameworks for implementing complex and flexible technologies like DTs. To recall, although we only picked a representative case study, DTs could be developed to represent *any* system of interest within the broader healthcare environment (16). Given this range of possibilities, general implementation frameworks could help anticipate and mitigate potential challenges, as illustrated in this work. Regarding the generalizability of these challenges, although this study is based on a specific case study, identified challenges are likely to apply to other DT use cases, and quite possibly to other high-tech implementation contexts that may lie at the intersection of AI, IoT, smart sensors, and decision-support (137, 138). Nevertheless, while we have a reasonable level of confidence in the relevance of these findings, their broader generalizability is contingent on further research.

Lastly, this study does not exhaustively document all DT implementation challenges due to our research design choices and the emerging nature of the DT literature. Our study relied on semi-structured interviews with a purposively selected group of stakeholders, which, while insightful, may not have captured the full spectrum of perspectives. It is plausible that many implementation issues remain unrecognized by both the DT community and our CFIR 2.0-guided analysis. Additionally, the findings are based on a single case study conducted within a family medicine clinic, which may limit their generalizability to other healthcare settings, particularly those with different organizational structures or resource constraints. Therefore, our findings should only be interpreted as a conservative estimate of the challenges involved in implementing DTs in HSs.

### 5.2 Points of departure from the literature

This study presents a significant point of departure from prior work by providing the first empirical assessment of CFIR 2.0's applicability in capturing DT implementation challenges within HSs, along with a comparison of these findings to the state of knowledge in the DT literature. As summarized in Table 5, previous research primarily relied on literature reviews (16, 82) and conceptual analyses (20) to examine DT adoption, while CFIR applications in healthcare employed qualitative methods in different interventions (139), with no focus on DT-specific implementations. In contrast, our study systematically applies CFIR 2.0 to a real-world DT implementation case in a family medicine clinic, validating its strengths and limitations using empirical data.

**Table 5:** A summary of points of departure from previous work (with representative references).

Criteria	Prior work on DT implementation in HS	Prior work on CFIR applications in HS	Contribution of this work
Focus	Concentrates on challenges in DT adoption through literature reviews (21, 82, 111) and conceptual analyses (20, 140)	Utilizes CFIR across various healthcare interventions (32, 35), yet lacks evaluation of DT technologies	The first empirical study applying CFIR 2.0 to a real-world DT implementation case in healthcare in its unique operational context
Methodological approach	Predominantly conceptual studies with minimal emphasis on practical implementation strategies (16)	Utilizes qualitative methods, including interviews and observations (35, 139, 141)	Employs semi-structured interviews and comparative analysis with literature to assess CFIR 2.0's empirical validity for DT adoption
Implementation frameworks used	Only a single study used NASSS framework to identify DT implementation challenges (82); other implementation science frameworks were not explored	Adapts CFIR constructs to fit specific healthcare contexts, such as patient-centered primary care (142), enhancing its relevance and applicability	First use of CFIR 2.0 to identify DT implementation challenges
Key challenges identified	Emphasizes technical challenges predominantly (16, 112)	Identifies institutional factors, like organizational culture and resource availability, that are critical to successful implementation of healthcare interventions (30, 143)	Identifies CFIR 2.0's efficacy in pinpointing organizational and financial barriers, while noting its limitations in addressing technological and data-centric issues specific to DTs
Integration of DT engineering and implementation science literature	Investigates implementation predominantly from an engineering standpoint, often overlooking behavioral and organizational factors that are critical for successful implementation (21)	Primarily centers on HS adoption challenges with limited consideration of engineering perspectives (144)	Bridges the gap between implementation science and DT engineering by comparing insights from both domains, facilitating cross-disciplinary knowledge transfer
Novelty	Discusses general DT adoption obstacles without employing a standardized evaluation framework (17, 21, 90)	Evaluates CFIR in various digital health interventions such as EHR (145), AI-assisted decision support (146), and telehealth platform (76), but does not explore DT implementation.	Presents a proof by contradiction to the implicit assumption that CFIR is an effective framework for assessing the implementation of complex technologies by documenting its shortcomings in capturing DT-specific challenges and proposing a need for adaptation

A key distinction lies in our methodological approach. Prior DT research is predominantly conceptual in nature, with minimal emphasis on the implementation side (16). Additionally, CFIR-based healthcare research lacked a DT-specific evaluation. Our study integrates semi-structured interviews with stakeholders and a comparative analysis with existing literature to assess CFIR 2.0's empirical validity. This enables a rich evaluation of its effectiveness in identifying organizational and financial barriers while revealing its shortcomings in capturing technological and data-related challenges that are fundamental to successful DT implementation in HSs.

Furthermore, Table 5 highlights *how* our study bridges the gap between engineering and implementation science perspectives. To elaborate, prior DT research that originates from the engineering community often overlooked behavioral and organizational factors (21). On the other hand, CFIR-based healthcare implementation studies had a very limited overview of engineering challenges, particularly those related to technologies such as DTs (144) that are considerably more complex (5) than let's say a simple diagnostic tool or a stand-alone algorithm to support some decision-making function (77, 146–149). Our findings challenge the implicit assumption that organizational factors dominate digital health adoption (150). Instead, we demonstrate that technological and data-centric challenges are equally critical, yet CFIR 2.0 fails to comprehensively capture them. This leads to our next point.

Lastly, this study provides a *proof by contradiction*, against the implicit assumption that CFIR is an effective framework to identify implementation challenges associated with high-tech, data-intensive new technologies (76, 151, 152). By documenting its limitations in guiding DT adoption, we highlight the need for an expanded CFIR model that integrates technological and data-specific constructs. This need aligns with the broader challenges identified in the recent NASEM report, which underscores foundational research gaps in DT development, including computational, statistical, and translational challenges that hinder their full realization (15). We anticipate that addressing these limitations will only become more pressing over time. As summarized in Table 5, this work provides a foundation for refining implementation science frameworks and DT adoption strategies, offering valuable insights for both digital health research and real-world DT deployment in HSs.

### 5.3 Theoretical implications

This study advances implementation science by critically examining how and to what extent CFIR 2.0 can identify DT implementation challenges within HSs. While CFIR 2.0 effectively captures organizational, financial, and sociotechnical barriers, it falls short in addressing critical technological and data-related issues. This limitation has significant theoretical implications, as it challenges the assumption that CFIR 2.0 is universally applicable to high-tech healthcare implementations. By documenting the nuanced set of factors that CFIR 2.0 omits, our study serves as a *proof by contradiction* to its claimed comprehensiveness, reinforcing the need for a more adaptable framework that integrates constructs tailored to digital health technologies.

By systematically contrasting empirical results with existing DT literature, this study demonstrates that technological and data-centric factors are not secondary concerns but fundamental to successful DT adoption. This finding challenges the prevailing assumption in the DT community that implementation challenges are predominantly organizational. By addressing this gap, our study bridges the disciplinary divide between implementation science and DT engineering, laying the groundwork for future interdisciplinary research on digital health technologies.

Methodologically, this study makes key contributions by applying CFIR 2.0 to a real-world case study, rather than relying on theoretical analysis alone, ensuring that the framework's applicability is assessed in an operational healthcare setting. Furthermore, our structured interviews with diverse stakeholders provide a richer and more nuanced dataset, capturing insights that would be missed in literature-based analyses.

Additionally, by identifying specific technological and data challenges that CFIR 2.0 fails to address, this study not only refines implementation science frameworks but also enhances DT literature by highlighting overlooked barriers. The rigorous comparison of CFIR 2.0's coverage against DT engineering literature ensures that findings are empirically grounded rather than assumed, setting a methodological precedent for future research.

### 5.4 Managerial implications

The findings of this study provide actionable insights for healthcare administrators, policymakers, and DT developers seeking to optimize implementation strategies; by outlining the nature of multi-faceted factors that (i) can be identified by the CFIR 2.0-guided interviews, (ii) will be missed if CFIR 2.0 is adopted as a guidance mechanism, and (iii) are identified both through CFIR 2.0 and in DT literature. Healthcare leaders can leverage our results to design evidence-based adoption frameworks that address both technical and social barriers, ensuring that DT initiatives align with broader institutional goals. Specifically, this study documents that fostering a culture of innovation readiness, integrating structured change management programs, and prioritizing stakeholder engagement is essential for successful implementation.

Additionally, DT developers and engineers must collaborate closely with IS experts to navigate non-technical barriers, such as workflow integration, training infrastructure, and user trust. Importantly, our findings also uncover key knowledge gaps and highlight opportunities for knowledge transfer, underscoring the potential for enhanced collaboration between the implementation science and DT communities. Such collaboration can drive the development of integrated frameworks and joint initiatives that address both technical and non-technical challenges.

Our study also highlights the importance of iterative evaluation mechanisms for the successful sustainment of DT technologies, ensuring that DTs are continuously adapted to the evolving needs of healthcare environments. These insights could serve as a strategic roadmap for managers to plan for, and execute, successful DT implementation in healthcare. We contend that the successful transition of this exciting technology into practice will remain a pressing challenge in years to come.

### 5.5 Future research directions

Future research could focus on refining the CFIR 2.0 framework to better capture the technological and data-related complexities inherent in DT implementation within HSs. Specifically, integrating constructs related to DT modeling and twinning, real-time data synchronization, sensor reliability, and data quality would enhance the framework's ability to address data-driven healthcare interventions. Longitudinal studies across diverse healthcare environments can be explored to validate the generalizability of these findings and to explore how organizational, technological, and sociotechnical dynamics evolve during DT adoption. Additionally, fostering interdisciplinary collaborations between the engineering and healthcare communities is essential for effectively bridging knowledge gaps. Such collaborative efforts can inform best practices for overcoming both technical and organizational barriers, thereby accelerating the transition of emerging technologies like DTs into practical healthcare solutions.

## 6 Conclusions

Within the field of implementation science, there have been calls to “specify and test mechanistic pathways… about drivers, moderators and mediators of implementation outcomes” and provide “finer-grained identification” of variables that influence and precede implementation outcomes (33). We sought to go beyond the use of CFIR 2.0 to better understand the organization and functioning of clinical microsystems and use it as a pre-implementation planning and evaluation tool for healthcare leaders and managers. Our study demonstrates the effectiveness of the CFIR 2.0 framework in identifying a significant portion of the challenges associated with DT implementation within HSs, while also highlighting its limitations in addressing technological and data-related issues.

As emphasized throughout this paper, the context is crucial for the development of accurate DTs, their seamless transition into practice, and sustained use by healthcare professionals. The framework's capacity to capture critical organizational and financial challenges that are commonly disregarded in the DT literature illustrates its value and points to an opportunity for informing the DT research community. By systematically bridging the knowledge gap between implementation science and DT communities, this study provides a foundation for interdisciplinary collaboration. The findings highlight how leveraging CFIR 2.0 can aid in identifying implementation barriers that extend beyond technical concerns, thereby facilitating a more holistic approach to DT adoption in healthcare. That being said, while CFIR is intended to be a universally applicable tool for any intervention in healthcare, the framework is deficient in capturing technological and data-related issues for emerging, complex, and data-intensive technologies such as DTs. This limitation underscores the necessity of refining existing implementation frameworks or developing hybrid models that integrate both sociotechnical and engineering considerations to ensure a more effective transition of DTs into clinical settings.

Beyond theoretical implications, these findings have practical significance for healthcare administrators and policymakers seeking to implement DTs in real-world settings. Understanding the multifaceted challenges identified in this study can inform the design of more effective strategies that align technological capabilities with organizational readiness, regulatory requirements, and stakeholder engagement. By fostering collaboration between the IS and DT communities, future research can explore structured pathways for optimizing DT implementation, thereby accelerating their impact on healthcare efficiency, provider well-being, and patient outcomes. Finally, this study reported insights for future DT implementations along with a foundation for enhancing CFIR 2.0's applicability to cutting-edge technologies across various healthcare contexts.

## Data Availability

The original contributions presented in the study are included in the article/Supplementary Material, further inquiries can be directed to the corresponding author.

## References

[1] Connor L Dean J McNett M Tydings DM Shrout A Gorsuch PF Evidence-based practice improves patient outcomes and healthcare system return on investment: findings from a scoping review. Worldviews Evid Based Nurs. (2023) 20(1):6–15. 10.1111/wvn.12621 36751881

[2] Vassolo RS Mac Cawley AF Tortorella GL Fogliatto FS Tlapa D Narayanamurthy G. Hospital investment decisions in healthcare 4.0 technologies: scoping review and framework for exploring challenges, trends, and research directions. J Med Internet Res. (2021) 23(8):e27571. 10.2196/27571 34435967 PMC8430851

[3] Carayon P. Human factors of complex sociotechnical systems. Appl Ergon. (2006) 37(4):525–35. 10.1016/j.apergo.2006.04.011 16756937

[4] Carayon P Hancock P Leveson N Noy I Sznelwar L Hootegem G van. Advancing a sociotechnical systems approach to workplace safety—developing the conceptual framework. Ergonomics. (2015) 58(4):548–64. 10.1080/00140139.2015.1015623 25831959 PMC4647652

[5] Hennig A Topcu TG Szajnfarber Z. So you think your system is complex? Why and how existing complexity measures rarely agree. J Mech Des. (2021) 144(4):041401. 10.1115/1.4052701

[6] Plsek PE Greenhalgh T. The challenge of complexity in health care. Br Med J. (2001) 323(7313):625–8. 10.1136/bmj.323.7313.625 11557716 PMC1121189

[7] Trochim WM Milstein B Wood BJ Jackson S Pressler V. Setting objectives for community and systems change: an application of concept mapping for planning a statewide health improvement initiative. Health Promot Pract. (2004) 5(1):8–19. 10.1177/1524839903258020 14965431

[8] Roy SN Shah BJ Gajjar H. Application of simulation in healthcare service operations: a review and research agenda. ACM Trans Model Comput Simul (TOMACS). (2020) 31(1):1–23. 10.1145/3427753

[9] Katsaliaki K Mustafee N. Applications of simulation within the healthcare context. J Oper Res Soc. (2011) 62(8):1431–51. 10.1057/jors.2010.20 32226177 PMC7099916

[10] Motola I Devine LA Chung HS Sullivan JE Issenberg SB. Simulation in healthcare education: a best evidence practical guide. AMEE guide No. 82. Med Teach. (2013) 35(10):e1511–30. 10.3109/0142159X.2013.818632 23941678

[11] Almagooshi S. Simulation modelling in healthcare: challenges and trends. Procedia Manuf. (2015) 3:301–7. 10.1016/j.promfg.2015.07.155

[12] Aaltonen P Ramaul L Kurvinen E Kutvonen A Nemeh A. Organizational barriers and enablers in reaching maturity in digital twin technology. In: Handbook of Digital Twins. Boca Raton, FL: CRC Press (2024). p. 386–400.

[13] Barn BS. The sociotechnical digital twin: on the gap between social and technical feasibility. *2022 IEEE 24th Conference on Business Informatics (CBI).* Amsterdam: IEEE (2022). p. 11–20.

[14] Rebentisch E Rhodes DH Soares AL Zimmerman R Tavares S. The digital twin as an enabler of digital transformation: a sociotechnical perspective. *2021 IEEE 19th International Conference on Industrial Informatics (INDIN).* Palma de Mallorca: IEEE (2021). p. 1–6.

[15] National Academies of Sciences E and Medicine. Foundational Research Gaps and Future Directions for Digital Twins. Washington, DC: National Academies Press (2023). 39088664

[16] Xames M Topcu TG. A systematic literature review of digital twin research for healthcare systems: research trends, gaps, and realization challenges. IEEE Access. (2024) 12:4099–126. 10.1109/ACCESS.2023.3349379

[17] Elkefi S Asan O. Digital twins for managing health care systems: rapid literature review. J Med Internet Res. (2022) 24(8):e37641. 10.2196/37641 35972776 PMC9428772

[18] Kaur MJ Mishra VP Maheshwari P. The convergence of digital twin, IoT, and machine learning: transforming data into action. In: Farsi M, et al., editors. Digital Twin Technologies and Smart Cities. Berlin/Heidelberg: Springer. (2020) 3–17.

[19] Rathore MM Shah SA Shukla D Bentafat E Bakiras S. The role of AI, machine learning, and big data in digital twinning: a systematic literature review, challenges, and opportunities. IEEE Access. (2021) 9:32030–52. 10.1109/ACCESS.2021.3060863

[20] Hassani H Huang X MacFeely S. Impactful digital twin in the healthcare revolution. Big Data Cogn Comput. (2022) 6(3):83. 10.3390/bdcc6030083

[21] Sun T He X Li Z. Digital twin in healthcare: recent updates and challenges. Digit Health. (2023) 9:20552076221149652. 10.1177/20552076221149651 PMC9830576 36636729

[22] Nundy S Cooper LA Mate KS. The quintuple aim for health care improvement: a new imperative to advance health equity. JAMA. (2022) 327(6):521–2. 10.1001/jama.2021.25181 35061006

[23] Sahal R Alsamhi SH Brown KN O’Shea D Alouffi B. Blockchain-based digital twins collaboration for smart pandemic alerting: decentralized COVID-19 pandemic alerting use case. Comput Intell Neurosci. (2022) 2022:786441. 10.1155/2022/7786441 PMC8759442 35035466

[24] Xames MD Topcu TG. Toward digital twins for human-in-the-loop systems: a framework for workload management and burnout prevention in healthcare systems. 2023 IEEE 3rd International Conference on Digital Twins and Parallel Intelligence (DTPI) (2023). p. 1–6

[25] Popa EO van Hilten M Oosterkamp E Bogaardt MJ. The use of digital twins in healthcare: socio-ethical benefits and socio-ethical risks. Life Sci Soc Policy. (2021) 17(1):6. 10.1186/s40504-021-00113-x 34218818 PMC8256511

[26] Song Y Li Y. Digital twin aided healthcare facility management: a case study of Shanghai Tongji Hospital. In: Jazizadeh F, et al., editors. Construction Research Congress 2022. Arlington, Virginia: American Society of Civil Engineers (2022). p. 1145–55. 10.1061/9780784483961.120

[27] Xames MD Topcu TG. How can digital twins support the economic, environmental, and social sustainability of healthcare systems: a systematic review focused on the triple-bottom-line. IEEE Access. (2025) 13:64390–411. 10.1109/ACCESS.2025.3559502

[28] Damschroder LJ Aron DC Keith RE Kirsh SR Alexander JA Lowery JC. Fostering implementation of health services research findings into practice: a consolidated framework for advancing implementation science. Implement Sci. (2009) 4(1):1–15. 10.1186/1748-5908-4-50 19664226 PMC2736161

[29] Stone AB Yuan CT Rosen MA Grant MC Benishek LE Hanahan E Barriers to and facilitators of implementing enhanced recovery pathways using an implementation framework: a systematic review. JAMA Surg. (2018) 153(3):270–9. 10.1001/jamasurg.2017.5565 29344622

[30] Varsi C Ekstedt M Gammon D Ruland CM. Using the consolidated framework for implementation research to identify barriers and facilitators for the implementation of an internet-based patient-provider communication service in five settings: a qualitative study. J Med Internet Res. (2015) 17(11):e262. 10.2196/jmir.5091 26582138 PMC4704938

[31] Rogers HL Pablo Hernando S Núñez-Fernández S Sanchez A Martos C Moreno M Barriers and facilitators in the implementation of an evidence-based health promotion intervention in a primary care setting: a qualitative study. J Health Organ Manag. (2021) 35(9):349–67. 10.1108/JHOM-12-2020-0512 PMC9136863 34464035

[32] Kirk MA Kelley C Yankey N Birken SA Abadie B Damschroder L. A systematic review of the use of the consolidated framework for implementation research. Implement Sci. (2015) 11:1–13. 10.1186/s13012-016-0437-z PMC4869309 27189233

[33] Proctor EK Bunger AC Lengnick-Hall R Gerke DR Martin JK Phillips RJ Ten years of implementation outcomes research: a scoping review. Implement Sci. (2023) 18(1):31. 10.1186/s13012-023-01286-z 37491242 PMC10367273

[34] van Tilburg ML Spin I Pisters MF Staal JB Ostelo RW van der Velde M Barriers and facilitators to the implementation of digital health services for people with musculoskeletal conditions in the primary health care setting: systematic review. J Med Internet Res. (2024) 26:e49868. 10.2196/49868 39190440 PMC11387918

[35] Damschroder LJ Reardon CM Widerquist MAO Lowery J. The updated consolidated framework for implementation research based on user feedback. Implement Sci. (2022) 17(1):75. 10.1186/s13012-022-01245-0 36309746 PMC9617234

[36] Grieves M Vickers J. Digital twin: mitigating unpredictable, undesirable emergent behavior in complex systems. In: Kahlen FJ Flumerfelt S Alves A, editors. Transdisciplinary Perspectives on Complex Systems: New Findings and Approaches. Cham: Springer International Publishing (2017). p. 85–113. 10.1007/978-3-319-38756-7_4

[37] Tao F Zhang M Liu Y Nee AYC. Digital twin driven prognostics and health management for complex equipment. CIRP Annals. (2018) 67(1):169–72. 10.1016/j.cirp.2018.04.055

[38] Lu Q Xie X Parlikad AK Schooling JM. Digital twin-enabled anomaly detection for built asset monitoring in operation and maintenance. Autom Constr. (2020) 118:103277. 10.1016/j.autcon.2020.103277

[39] van Dinter R Tekinerdogan B Catal C. Predictive maintenance using digital twins: a systematic literature review. Inf Softw Technol. (2022) 151:107008. 10.1016/j.infsof.2022.107008

[40] Thelen A Zhang X Fink O Lu Y Ghosh S Youn BD A comprehensive review of digital twin—part 1: modeling and twinning enabling technologies. Struct Multidiscipl Optim. (2022) 65(12):354. 10.1007/s00158-022-03425-4

[41] Vallée A. Digital twin for healthcare systems. Front Digit Health. (2023) 5:1253050. 10.3389/fdgth.2023.1253050 37744683 PMC10513171

[42] Vallée A. Envisioning the future of personalized medicine: role and realities of digital twins. J Med Internet Res. (2024) 26:e50204. 10.2196/50204 38739913 PMC11130780

[43] Barricelli BR Casiraghi E Gliozzo J Petrini A Valtolina S. Human digital twin for fitness management. IEEE Access. (2020) 8:26637–64. 10.1109/ACCESS.2020.2971576

[44] Béthencourt L Dabachine W Dejouy V Lalmiche Z Neuberger K Ibnouhsein I Guiding measurement protocols of connected medical devices using digital twins: a statistical methodology applied to detecting and monitoring lymphedema. IEEE Access. (2021) 9:39444–65. 10.1109/ACCESS.2021.3063786

[45] Uhlenberg L Derungs A Amft O. Co-simulation of human digital twins and wearable inertial sensors to analyse gait event estimation. Front Bioeng Biotechnol. (2023) 11:1104000. 10.3389/fbioe.2023.1104000 37122859 PMC10132030

[46] Chude-Okonkwo UK. Conceptual molecular communication solution for developing digital twin to enable precision medicine implementation. In: 2021 15th International Conference on Signal Processing and Communication Systems (ICSPCS). IEEE (2021). p. 1–10.

[47] Loewe A Martínez Díaz P Nagel C Sánchez J. Cardiac digital twin modeling. In: Jadczyk T, et al., editors. Innovative Treatment Strategies for Clinical Electrophysiology. Singapore: Springer (2022). p. 111–34.

[48] Venkatapurapu SP Gibbs M Kimko H. Augmented intelligence in precision medicine: transforming clinical decision-making with AI/ML and/or quantitative systems pharmacology models. Clin Transl Sci. (2024) 17(12):e70112. 10.1111/cts.70112 39673165 PMC11645444

[49] Katsoulakis E Wang Q Wu H Shahriyari L Fletcher R Liu J Digital twins for health: a scoping review. NPJ Digit Med. (2024) 7(1):77. 10.1038/s41746-024-01073-0 38519626 PMC10960047

[50] Kobayashi T Fukae K Imai T Arai K. Dementia sign detection system using digital twin. 2021 Ninth International Symposium on Computing and Networking (CANDAR) (2021). p. 127–33

[51] Khan S Saied IM Ratnarajah T Arslan T. Evaluation of unobtrusive microwave sensors in healthcare 4.0-toward the creation of digital-twin model. Sensors. (2022) 22(21):8519. 10.3390/s22218519 36366218 PMC9657877

[52] Wei H. A method for patient gait real-time monitoring based on powered exoskeleton and digital twin. In: Shaozi L, Zhiyong Y, editors. *Proceedings of SPIE, Volume 12566, Fifth International Conference on Computer Information Science and Artificial Intelligence (CISAI 2022)*. Arlington, VA: SPIE (2023). p. 125662U. 10.1117/12.2667794

[53] Qin Y Ma M Shen L Wang H Han J. Virtual and real bidirectional driving system for the synchronization of manipulations in robotic joint surgeries. Machines. (2022) 10(7):530. 10.3390/machines10070530

[54] Shu H Liang R Li Z Goodridge A Zhang X Ding H Twin-S: a digital twin for skull base surgery. Int J Comput Assist Radiol Surg. (2023) 18(6):1077–84. 10.1007/s11548-023-02863-9 37160583 PMC11110948

[55] Moztarzadeh O Jamshidi M Sargolzaei S Keikhaee F Jamshidi A Shadroo S Metaverse and medical diagnosis: a blockchain-based digital twinning approach based on MobileNetV2 algorithm for cervical vertebral maturation. Diagnostics. (2023) 13(8):1485. 10.3390/diagnostics13081485 37189587 PMC10137959

[56] Shibuya S Shido N Shirai R Sase K Ebina K Chen X Proposal of simulation-based surgical navigation and development of laparoscopic surgical simulator that reflects motion of surgical instruments in real-world. Int J Autom Technol. (2023) 17(3):262–76. 10.20965/ijat.2023.p0262

[57] Hu X Cao H Shi J Dai Y Dai W. Study of hospital emergency resource scheduling based on digital twin technology. 2021 IEEE 2nd International Conference on Information Technology, Big Data and Artificial Intelligence (ICIBA) (2021). p. 1059–63

[58] Karakra A Lamine E Fontanili F Lamothe J. Hospit’win: a digital twin framework for patients’ pathways real-time monitoring and hospital organizational resilience capacity enhancement. In: Proceedings of the 9th International Workshop on Innovative Simulation for Healthcare (IWISH 2020). CAL-TEK srl (2020). p. 62–71. Available online at: https://www.cal-tek.eu/proceedings/i3m/2020/iwish/012 (Accessed September 10, 2023).

[59] Augusto V Murgier M Viallon A. A modelling and simulation framework for in℡ligent control of emergency units in the case of Major crisis. In: Johansson B, et al., editors. 2018 Winter Simulation Conference (WSC). Gothenburg: IEEE Press (2018). p. 2495–506.

[60] Bouleux G El Haouzi HB Cheutet V Demesure G Derigent W Moyaux T Requirements for a digital twin for an emergency department. In: Borangiu T Trentesaux D Leitão P, editors. Service Oriented, Holonic and Multi-Agent Manufacturing Systems for Industry of the Future. Cham: Springer International Publishing (2023). p. 130–41.

[61] Karakra A Fontanili F Lamine E Lamothe J Taweel A. Pervasive computing integrated discrete event simulation for a hospital digital twin. In: 2018 IEEE/ACS 15th International Conference on Computer Systems and Applications (AICCSA). (2018). p. 1–6.

[62] Liu Y Zhang L Yang Y Zhou L Ren L Wang F A novel cloud-based framework for the elderly healthcare services using digital twin. IEEE Access. (2019) 7:49088–101. 10.1109/ACCESS.2019.2909828

[63] Spitzer M Dattner I Zilcha-Mano S. Digital twins and the future of precision mental health. Front Psychiatry. (2023) 14:1082598. 10.3389/fpsyt.2023.1082598 36993921 PMC10040602

[64] An G Cockrell C. Drug development digital twins for drug discovery, testing and repurposing: a schema for requirements and development. Front Syst Biol. (2022) 2:928387. 10.3389/fsysb.2022.928387 35935475 PMC9351294

[65] Alrashed S Min-Allah N Ali I Mehmood R. COVID-19 outbreak and the role of digital twin. Multimed Tools Appl. (2022) 81(19):26857–71. 10.1007/s11042-021-11664-8 35002471 PMC8721629

[66] Carayon P Hoonakker P. Human factors and usability for health information technology: old and new challenges. Yearb Med Inform. (2019) 28(01):71–7. 10.1055/s-0039-1677907 31419818 PMC6697515

[67] Ratwani RM Reider J Singh H. A decade of health information technology usability challenges and the path forward. JAMA. (2019) 321(8):743–4. 10.1001/jama.2019.0161 30715133

[68] Covvey HD. Healthcare as a complex adaptive system. In: van Gemert-Pijnen L, et al., editors. EHealth Research, Theory and Development. Abingdon: Routledge (2018). p. 69–90.

[69] Nilsen P. Making sense of implementation theories, models, and frameworks. Implement Sci. (2020) 30:53–79. 10.1007/978-3-030-03874-8_3 PMC4406164 25895742

[70] Glasgow RE Vogt TM Boles SM. Evaluating the public health impact of health promotion interventions: the RE-AIM framework. Am J Public Health. (1999) 89(9):1322–7. 10.2105/AJPH.89.9.1322 10474547 PMC1508772

[71] Kitson AL Rycroft-Malone J Harvey G McCormack B Seers K Titchen A. Evaluating the successful implementation of evidence into practice using the PARiHS framework: theoretical and practical challenges. Implement Sci. (2008) 3:1–12. 10.1186/1748-5908-3-1 18179688 PMC2235887

[72] Greenhalgh T Wherton J Papoutsi C Lynch J Hughes G Hinder S Beyond adoption: a new framework for theorizing and evaluating nonadoption, abandonment, and challenges to the scale-up, spread, and sustainability of health and care technologies. J Med Internet Res. (2017) 19(11):e8775. 10.2196/jmir.8775 PMC5688245 29092808

[73] Bergström A Ehrenberg A Eldh AC Graham ID Gustafsson K Harvey G The use of the PARIHS framework in implementation research and practice—a citation analysis of the literature. Implement Sci. (2020) 15:1–51. 10.1186/s13012-020-01003-0 32854718 PMC7450685

[74] Shin HD Hamovitch E Gatov E MacKinnon M Samawi L Boateng R The NASSS (Non-Adoption, Abandonment, Scale-Up, Spread and Sustainability) framework use over time: a scoping review. *PLOS Digit. Health*. (2025) 4(3):e0000418. 10.1371/journal.pdig.0000418 PMC11913280 40096260

[75] Villalobos Dintrans P Bossert TJ Sherry J Kruk ME. A synthesis of implementation science frameworks and application to global health gaps. Global Health Res Policy. (2019) 4(1):1–11. 10.1186/s41256-019-0115-1 PMC6712702 31485483

[76] Rangachari P Mushiana SS Herbert K. A scoping review of applications of the consolidated framework for implementation research (CFIR) to telehealth service implementation initiatives. BMC Health Serv Res. (2022) 22(1):1450. 10.1186/s12913-022-08871-w 36447279 PMC9708146

[77] Liao X Yao C Jin F Zhang J Liu L. Barriers and facilitators to implementing imaging-based diagnostic artificial intelligence-assisted decision-making software in hospitals in China: a qualitative study using the updated consolidated framework for implementation research. BMJ Open. (2024) 14(9):e084398. 10.1136/bmjopen-2024-084398 39260855 PMC11409362

[78] Lyu J Zhang H Wang H Liu X Jing Y Yin L Facilitators and barriers to implementing patient-reported outcomes in clinical oncology practice: a systematic review based on the consolidated framework for implementation research. Implement Sci Commun. (2024) 5(1):120. 10.1186/s43058-024-00654-0 39473015 PMC11520578

[79] Bourke JA Jerram KAS Arora M Craig A Middleton JW. Using the consolidated framework for implementation research to integrate innovation recipients’ perspectives into the implementation of a digital version of the spinal cord injury health maintenance tool: a qualitative analysis. BMC Health Serv Res. (2024) 24(1):390. 10.1186/s12913-024-10847-x 38549148 PMC10976821

[80] Skolarus TA Lehmann T Tabak RG Harris J Lecy J Sales AE. Assessing citation networks for dissemination and implementation research frameworks. Implement Sci. (2017) 12:1–17. 10.1186/s13012-017-0628-2 28754140 PMC5534119

[81] National Academies of Sciences E. Taking Action Against Clinician Burnout: A Systems Approach to Professional Well-Being. Washington, DC: National Academies Press (2019). 31940160

[82] Winter PD Chico TJ. Using the non-adoption, abandonment, scale-up, spread, and sustainability (NASSS) framework to identify barriers and facilitators for the implementation of digital twins in cardiovascular medicine. Sensors. (2023) 23(14):6333. 10.3390/s23146333 37514627 PMC10385429

[83] Starfield B Shi L Macinko J. Contribution of primary care to health systems and health. Milbank Q. (2005) 83(3):457–502. 10.1111/j.1468-0009.2005.00409.x 16202000 PMC2690145

[84] Lilly CM Cucchi E Marshall N Katz A. Battling intensivist burnout: a role for workload management. Chest. (2019) 156(5):1001–7. 10.1016/j.chest.2019.04.103 31102610

[85] Patel RS Sekhri S Bhimanadham NN Imran S Hossain S. A review on strategies to manage physician burnout. Cureus. (2019) 11(6):e4805. 10.7759/cureus.4805 31404361 PMC6682395

[86] Nelson EC Batalden PB Godfrey MM. Quality by Design: A Clinical Microsystems Approach. Hoboken, NJ: John Wiley & Sons (2011).

[87] Shi L. The impact of primary care: a focused review. Scientifica (Cairo). (2012) 2012(1):432892. 10.6064/2012/432892 24278694 PMC3820521

[88] Bureau of Health Workforce. State of the Primary Care Workforce 2023. Health Resources and Services Administration (2023). Available online at: https://bhw.hrsa.gov/sites/default/files/bureau-health-workforce/data-research/state-of-primary-care-workforce-2023.pdf

[89] Kane L. Medscape physician burnout & depression report 2022: stress, anxiety, and anger. Medscape. (2022).

[90] Xames MD Topcu TG. A rapid review of how model-based systems engineering is used in healthcare systems. INCOSE International Symposium. (2024) 34(1):1477–62. 10.1002/iis2.13218

[91] Blanchard BS Fabrycky WJ Fabrycky WJ. Systems Engineering and Analysis. Vol. 4. Englewood Cliffs, NJ: Prentice hall (1990).

[92] Hennink M Kaiser BN. Sample sizes for saturation in qualitative research: a systematic review of empirical tests. Soc Sci Med. (2022) 292:114523. 10.1016/j.socscimed.2021.114523 34785096

[93] Cellina M Cè M Alì M Irmici G Ibba S Caloro E Digital twins: the new frontier for personalized medicine? Appl Sci. (2023) 13(13):7940. 10.3390/app13137940

[94] Mohamed N Al-Jaroodi J Jawhar I Kesserwan N. Leveraging digital twins for healthcare systems engineering. Ieee Access. (2023) 11:69841–53. 10.1109/ACCESS.2023.3292119

[95] Alazab M Khan LU Koppu S Ramu SP Iyapparaja M Boobalan P Digital twins for healthcare 4.0—recent advances, architecture, and open challenges. IEEE Consum Electroni Mag. (2022) 12(6):29–37. 10.1109/MCE.2022.3208986

[96] Elayan H Aloqaily M Guizani M. Digital twin for intelligent context-aware IoT healthcare systems. IEEE Internet Things J. (2021) 8(23):16749–57. 10.1109/JIOT.2021.3051158

[97] Dahir H Luna J Khattab A Abrougui K Kumar R. Challenges of digital twin in healthcare. In: El Saddik A, editor. Digital Twin for Healthcare. Amsterdam: Elsevier (2023). p. 73–95.

[98] Chu Y Li S Tang J Wu H. The potential of the medical digital twin in diabetes management: a review. Front Med (Lausanne). (2023) 10:1178912. 10.3389/fmed.2023.1178912 37547605 PMC10397506

[99] Jimenez JI Jahankhani H Kendzierskyj S. Health care in the cyberspace: medical cyber-physical system and digital twin challenges. In: Farsi M, et al., editors. Digital Twin Technologies and Smart Cities. Heidelberg: Springer. (2020). p. 79–92. 10.1007/978-3-030-18732-3_6

[100] Melesse TY Di Pasquale V Riemma S. Digital twin models in industrial operations: a systematic literature review. Procedia Manuf. (2020) 42:267–72. 10.1016/j.promfg.2020.02.084

[101] Arin IA Warnars HL Murad DF. A systematic literature review of recent trends and challenges in digital twin implementation. *2023 10th International Conference on ICT for Smart Society (ICISS).* Bandung: IEEE (2023). p. 1–10.

[102] Perno M Hvam L Haug A. Implementation of digital twins in the process industry: a systematic literature review of enablers and barriers. Comput Ind. (2022) 134:103558. 10.1016/j.compind.2021.103558

[103] Turab M Jamil S. A comprehensive survey of digital twins in healthcare in the era of metaverse. BioMedInformatics. (2023) 3(3):563–84. 10.3390/biomedinformatics3030039

[104] Haleem A Javaid M Singh RP Suman R. Exploring the revolution in healthcare systems through the applications of digital twin technology. Biomed Technol. (2023) 4:28–38. 10.1016/j.bmt.2023.02.001

[105] Subasi A Subasi ME. Digital twins in healthcare and biomedicine. In: de Pablos Heredero P, Ifeachor E, editors. Artificial Intelligence, Big Data, Blockchain and 5G for the Digital Transformation of the Healthcare Industry. Amsterdam: Elsevier (2024). p. 365–401.

[106] Barricelli BR Casiraghi E Fogli D. A survey on digital twin: definitions, characteristics, applications, and design implications. IEEE Access. (2019) 7:167653–71. 10.1109/ACCESS.2019.2953499

[107] Vallée A. Challenges and directions for digital twin implementation in otorhinolaryngology. Eur Arch Otorhinolaryngol. (2024) 281(11):6155–9. 10.1007/s00405-024-08662-5 38703196

[108] Corral-Acero J Margara F Marciniak M Rodero C Loncaric F Feng Y The “Digital Twin” to enable the vision of precision cardiology. Eur Heart J. (2020) 41(48):4556–64. 10.1093/eurheartj/ehaa159 32128588 PMC7774470

[109] Coorey G Figtree GA Fletcher DF Snelson VJ Vernon ST Winlaw D The health digital twin to tackle cardiovascular disease—a review of an emerging interdisciplinary field. NPJ Digit Med. (2022) 5(1):126. 10.1038/s41746-022-00640-7 36028526 PMC9418270

[110] Lutze R. Digital twins in eHealth–: prospects and challenges focussing on information management. *2019 IEEE international conference on engineering, technology and innovation (ICE/ITMC).* Valbonne Sophia-Antipolis: IEEE (2019). p. 1–9.

[111] Meijer C Uh HW El Bouhaddani S. Digital twins in healthcare: methodological challenges and opportunities. J Pers Med. (2023) 13(10):1522. 10.3390/jpm13101522 37888133 PMC10608065

[112] Sun T He X Song X Shu L Li Z. The digital twin in medicine: a key to the future of healthcare? Front Med (Lausanne). (2022) 9:907066. 10.3389/fmed.2022.907066 35911407 PMC9330225

[113] Aloqaily M Bouachir O Karray F. Digital twin for healthcare immersive services: fundamentals, architectures, and open issues. In: Ghrayeb RN, et al., editors. Digital Twin for Healthcare. Amsterdam: Elsevier (2023). p. 39–71.

[114] Machado TM Berssaneti FT. Literature review of digital twin in healthcare. Heliyon. (2023) 9(9):e19390. 10.1016/j.heliyon.2023.e19390 37809792 PMC10558347

[115] Das C Mumu AA MdF A Sarker SK Muyeen SM Das SK Toward IoRT collaborative digital twin technology enabled future surgical sector: technical innovations, opportunities and challenges. IEEE Access. (2022) 10:129079–104. 10.1109/ACCESS.2022.3227644

[116] Drummond D Coulet A. Technical, ethical, legal, and societal challenges with digital twin systems for the management of chronic diseases in children and young people. J Med Internet Res. (2022) 24(10):e39698. 10.2196/39698 36315239 PMC9664337

[117] Braun M. Represent me: please! towards an ethics of digital twins in medicine. J Med Ethics. (2021) 47(6):394–400. 10.1136/medethics-2020-106134 33722986

[118] Bruynseels K Santoni de Sio F Van den Hoven J. Digital twins in health care: ethical implications of an emerging engineering paradigm. Front Genet. (2018) 9:31. 10.3389/fgene.2018.00031 29487613 PMC5816748

[119] Huang PH Kim KH Schermer M. Ethical issues of digital twins for personalized health care service: preliminary mapping study. J Med Internet Res. (2022) 24(1):e33081. 10.2196/33081 35099399 PMC8844982

[120] Iqbal JD Krauthammer M Biller-Andorno N. The use and ethics of digital twins in medicine. J Law Med Ethics. (2022) 50(3):583–96. 10.1017/jme.2022.97 36398633

[121] Kamel Boulos MN Zhang P. Digital twins: from personalised medicine to precision public health. J Pers Med. (2021) 11(8):745. 10.3390/jpm11080745 34442389 PMC8401029

[122] Trauer J Mutschler M Mörtl M Zimmermann M. Challenges in implementing digital twins–a survey. *International Design Engineering Technical Conferences and Computers and Information in Engineering Conference 2022 Aug 14*. St. Louis, MO: American Society of Mechanical Engineers (2022). Vol. 86212, p. V002T02A055.

[123] VanDerHorn E Mahadevan S. Digital twin: generalization, characterization and implementation. Decis Support Syst. (2021) 145:113524. 10.1016/j.dss.2021.113524

[124] Fuller A Fan Z Day C Barlow C. Digital twin: enabling technologies, challenges and open research. IEEE Access. (2020) 8:108952–71. 10.1109/ACCESS.2020.2998358

[125] Zheng X Lu J Kiritsis D. The emergence of cognitive digital twin: vision, challenges and opportunities. Int J Product Res. (2022) 60(24):7610–32. 10.1080/00207543.2021.2014591

[126] Tripathi N Hietala H Xu Y Liyanage R. Stakeholders collaborations, challenges and emerging concepts in digital twin ecosystems. Inf Softw Technol. (2024) 169:107424. 10.1016/j.infsof.2024.107424

[127] Jangoan S Krishnamoorthy G Muthusubramanian M Sharma KK. Demystifying explainable AI: understanding, transparency, and trust. Int J Multidiscip Res. (2024) 6(2):1–13. 10.36948/ijfmr.2024.v06i02.14597

[128] Al Alawi S Al Dhaheri A Al Baloushi D Al Dhaheri M Prinsloo EA. Physician user satisfaction with an electronic medical records system in primary healthcare centres in Al Ain: a qualitative study. BMJ Open. (2014) 4(11):e005569. 10.1136/bmjopen-2014-005569 25377010 PMC4225459

[129] Nguyen L Bellucci E Nguyen LT. Electronic health records implementation: an evaluation of information system impact and contingency factors. Int J Med Inf. (2014) 83(11):779–96. 10.1016/j.ijmedinf.2014.06.011 25085286

[130] Unni P Staes C Weeks H Kramer H Borbolla D Slager S Why aren’t they happy? An analysis of end-user satisfaction with electronic health records. *AMIA Annual Symposium Proceedings 2017 Feb 10.* Washington, DC: American Medical Informatics Association (2016). Vol. 2016, p. 2026. PMC5333231 28269962

[131] Salado A Kannan H. Elemental patterns of verification strategies. Syst Eng. (2019) 22(5):370–88. 10.1002/sys.21481

[132] Liu Y Ong S Nee A. State-of-the-art survey on digital twin implementations. Adv Manuf. (2022) 10(1):1–23. 10.1007/s40436-021-00375-w

[133] Venkatapurapu SP DeWitt MT Behar M D’Alessandro PM. Digital twins for proactive and personalized healthcare–challenges and opportunities. In: Lyu Z, editor. *Handbook of Digital Twins*. 1st ed. Boca Raton, FL: CRC Press (2024). p. 888–902. 10.1201/9781003425724-61

[134] Sage AP Cuppan CD. On the systems engineering and management of systems of systems and federations of systems. Inform Knowl Syst Manag. (2001) 2(4):325–45. 10.3233/IKS-2001-00045

[135] Kossiakoff A Biemer SM Seymour SJ Flanigan DA. Systems Engineering Principles and Practice. Hoboken, NJ: John Wiley & Sons (2020).

[136] Prescient & Strategic Intelligence. Digital Twin Market Report—Global Industry Growth and Demand Forecast to 2030. Noida, Uttar Pradesh: PS Market Research (2022). Report No.: 11941. Available online at: https://alertable.fidelity.com/ftgw/alerts/GetAlertsSummary (Accessed August 22, 2023).

[137] Chataut R Phoummalayvane A Akl R. Unleashing the power of IoT: a comprehensive review of IoT applications and future prospects in healthcare, agriculture, smart homes, smart cities, and industry 4.0. Sensors. (2023) 23(16):7194. 10.3390/s23167194 37631731 PMC10458191

[138] Bajic B Rikalovic A Suzic N Piuri V. Industry 4.0 implementation challenges and opportunities: a managerial perspective. IEEE Syst J. (2020) 15(1):546–59. 10.1109/JSYST.2020.3023041

[139] Nevedal AL Reardon CM Widerquist O Jackson MA Cutrona GL White SL Rapid versus traditional qualitative analysis using the consolidated framework for implementation research (CFIR). Implement Sci. (2021) 16(1):67. 10.1186/s13012-021-01111-5 34215286 PMC8252308

[140] Okegbile SD Cai J Niyato D Yi C. Human digital twin for personalized healthcare: vision, architecture and future directions. IEEE Netw. (2023) 37(2):262–9. 10.1109/MNET.118.2200071

[141] Warner G Lawson B Sampalli T Burge F Gibson R Wood S. Applying the consolidated framework for implementation research to identify barriers affecting implementation of an online frailty tool into primary health care: a qualitative study. BMC Health Serv Res. (2018) 18:1–11. 10.1186/s12913-018-3163-1 29855306 PMC5984376

[142] Safaeinili N Brown-Johnson C Shaw JG Mahoney M Winget M. CFIR simplified: pragmatic application of and adaptations to the consolidated framework for implementation research (CFIR) for evaluation of a patient-centered care transformation within a learning health system. Learn Health Syst. (2020) 4(1):e10201. 10.1002/lrh2.10201 31989028 PMC6971122

[143] Li SA Jeffs L Barwick M Stevens B. Organizational contextual features that influence the implementation of evidence-based practices across healthcare settings: a systematic integrative review. Syst Rev. (2018) 7:1–19. 10.1186/s13643-018-0734-5 29729669 PMC5936626

[144] Birken SA Powell BJ Presseau J Kirk MA Lorencatto F Gould NJ Combined use of the consolidated framework for implementation research (CFIR) and the theoretical domains framework (TDF): a systematic review. Implement Sci. (2017) 12:1–14. 10.1186/s13012-016-0534-z 28057049 PMC5217749

[145] Richardson JE Abramson EL Pfoh ER Kaushal R, HITEC Investigators. Bridging informatics and implementation science: evaluating a framework to assess electronic health record implementations in community settings. *AMIA Annual Symposium Proceedings 2012 Nov 3*. Seattle, Washington: American Medical Informatics Association (2012). Vol. 2012, p. 770. PMC3540540 23304351

[146] Finkelstein J Gabriel A Schmer S Truong TT Dunn A. Identifying facilitators and barriers to implementation of AI-assisted clinical decision support in an electronic health record system. J Med Syst. (2024) 48(1):89. 10.1007/s10916-024-02104-9 39292314 PMC11410896

[147] Pratt R Saman DM Allen C Crabtree B Ohnsorg K Sperl-Hillen JM Assessing the implementation of a clinical decision support tool in primary care for diabetes prevention: a qualitative interview study using the consolidated framework for implementation science. BMC Med Inform Decis Mak. (2022) 22(1):15. 10.1186/s12911-021-01745-x 35033029 PMC8760770

[148] Sung M He J Zhou Q Chen Y Ji JS Chen H Using an integrated framework to investigate the facilitators and barriers of health information technology implementation in noncommunicable disease management: systematic review. J Med Internet Res. (2022) 24(7):e37338. 10.2196/37338 35857364 PMC9350822

[149] Ho V Johnson CB Ghanzouri I Amal S Asch S Ross E. Physician-and patient-elicited barriers and facilitators to implementation of a machine learning–based screening tool for peripheral arterial disease: preimplementation study with physician and patient stakeholders. JMIR Cardio. (2023) 7(1):e44732. 10.2196/44732 37930755 PMC10660241

[150] Brommeyer M Whittaker M Liang Z. Organizational factors driving the realization of digital health transformation benefits from health service managers: a qualitative study. J Healthc Leadersh. (2024) 16:455–72. 10.2147/JHL.S487589 39524481 PMC11546275

[151] King DK Shoup JA Raebel MA Anderson CB Wagner NM Ritzwoller DP Planning for implementation success using RE-AIM and CFIR frameworks: a qualitative study. Front Public Health. (2020) 8:59. 10.3389/fpubh.2020.00059 32195217 PMC7063029

[152] Mosch LK Poncette AS Spies C Weber-Carstens S Schieler M Krampe H Creation of an evidence-based implementation framework for digital health technology in the intensive care unit: qualitative study. JMIR formative Research. (2022) 6(4):e22866. 10.2196/22866 35394445 PMC9034425

