# Supplementary material for: Evaluating CFIR 2.0 in identifying digital twin implementation challenges in healthcare: bridging the dichotomy between engineering and healthcare communities
Source: Front Digit Health. 2025 Sep 15;7:1611225. doi: 10.3389/fdgth.2025.1611225 (PMC12477195; doi:10.3389/fdgth.2025.1611225)
Supplement: Supplementary file 1 [file Table1.docx]

***Supplementary material***

**Supplementary table**

**Supplementary Table 1.** Challenges identified through CFIR 2.0-guided interviews of different stakeholders for our DT case study.

| **CFIR 2.0 Domain** | **Challenges identified** | **Stakeholders who identified the challenge** |
| --- | --- | --- |
| Innovation | Trust in DT developers/vendors | OP, EE, FMS, IS |
|  | Lack of prior research/Lack of evidence on human-in-the-loop systems | OP, FMS, EE |
|  | High initial cost/Financial viability concerns | OP, EE, IS |
|  | Validation and verification | EE, FMS, IS |
|  | Testing and evaluation | EE, FMS, IS |
|  | High real-time data needs | EE, FMS, IS |
|  | Interoperability/Integration complexity | EE, IS |
|  | Lack of generalizability | EE, IS |
|  | Lack of standardization | EE |
|  | User-specific personalization | EE |
|  | Usability issues | EE |
|  | Data integration/fusion | EE |
|  | Scope/adaptability management in a dynamic landscape | FMS |
|  | Unclear/intangible benefits | FMS |
|  | Need for integrated decision support systems | FMS |
|  | Human-work interaction design | FMS |
|  | Free trialability | FMS |
|  | ROI uncertainty | FMS |
| Outer setting | Data privacy | EE, FMS, IS, OP |
|  | Data security | FMS, EE, IS, OP |
|  | Ethical issues | EE, FMS, IS, OP |
|  | Willingness for upfront investment | OP, EE, FMS |
|  | Organizational inertia/resistance to change | EE, OP, IS |
|  | Organization's technology readiness | EE, OP, IS |
|  | Data accessibility | OP, FMS, EE |
|  | Collaboration and communication barriers | OP, EE, IS |
|  | Legal issues | EE, FMS, IS |
|  | Regulatory compliance | EE, FMS, IS |
|  | Providers’ perception on the technology/Technophobia among older generation providers | EE, OP |
|  | Unclear/intangible benefits | FMS, IS |
|  | Lack of established best practice guidelines | OP |
|  | Pressure for immediate results | OP |
|  | DT adaptability | EE |
|  | Key performance indicators (KPIs) tracking and management | EE |
|  | Resilience to low-frequency, high-impact disruptions | FMS |
|  | Alignment with organizational goals and stakeholder perceptions | FMS |
|  | Time-scale dependency of DT performance | FMS |
|  | Staffing shortage | FMS |
|  | Providers’ perception on the technology/Technophobia among older generation providers | FMS |
|  | Fear of added workload | FMS |
|  | Collaboration and communication barriers | FMS |
|  | Misalignment of outcomes and incentives | FMS |
| Inner setting | Unclear/intangible benefits | OP, EE, FMS, IS |
|  | Collaboration and communication barriers | OP, EE, IS |
|  | Interoperability | EE, FMS, IS |
|  | Quantification of benefits and outcomes | OP, FMS |
|  | Lack of training and support infrastructure | OP, EE |
|  | Misalignment of outcomes and incentives | FMS, IS |
|  | Reflecting stakeholders’ needs/Stakeholder engagement | OP, IS |
|  | Individual resistance to change | FMS, IS |
|  | Trust and transparency in implementation | IS |
|  | Infrastructure and workflow integration | OP |
|  | Providers’ perception on the technology/Technophobia among older generation providers | OP |
|  | Reversibility of operational decisions | OP |
|  | Data governance | OP |
|  | Scalability | EE |
|  | Identification of the need/problem | EE |
|  | Organization’s technology readiness | EE |
|  | Model latency and timeliness | FMS |
|  | Data fragmentation/Siloed data sources | FMS |
|  | Cultural emphasis on reactive work and reporting over foundational system issues | FMS |
|  | Lack of immediate benefits | FMS |
|  | Differences in leadership styles | FMS |
|  | Alignment with organizational goals and stakeholder perceptions | FMS |
|  | ROI uncertainty | FMS |
|  | Availability of resources to implement operational changes | FMS |
|  | Need for integrated decision support systems | FMS |
| Individuals | Individual’s inertia/resistance to change | EE, IS, FMS |
|  | Collaboration and communication barriers | EE, IS |
|  | Individual’s commitment to implementation | EE, IS |
|  | Differences in motivation levels among individuals/groups | FMS, IS |
|  | Identification of the need/problem | EE, IS |
|  | Lack of understanding of the technology | EE, IS |
|  | Usability issues | EE |
|  | Unclear/intangible benefits | EE |
|  | Lack of expertise | FMS |
|  | Presence of shadow influencers | FMS |
|  | Lack of understanding of perceived benefits | FMS |
|  | Provider schedule constraints for technology learning | FMS |
| Implementation process | Identification of the need/problem | OP, EE, FMS, IS |
|  | Collaboration and communication barriers | EE, FMS, IS |
|  | Scope management in a dynamic landscape | EE, FMS, IS |
|  | Individual’s commitment to implementation | EE, FMS |
|  | Effective reflection, evaluation, and feedback mechanisms | FMS, IS |
|  | Value communication | OP |
|  | Alignment with organizational goals and stakeholder perceptions | OP |
|  | KPI tracking and management | EE |
|  | Interoperability | EE |
|  | Performance concerns | FMS |
|  | Change fatigue | FMS |
|  | Data ownership | FMS |
|  | Validation and verification | FMS |
|  | Pressure for immediate results | FMS |

*OP=Organizational psychologists; IS=Implementation scientists; FMS=Family medicine specialists; EE=Engineers
